# Construction and validation of an information portal on combined HIV prevention*

**DOI:** 10.1590/1518-8345.7221.4509

**Published:** 2025-05-02

**Authors:** Felipe Martins Lioi, Laelson Rochelle Milanês Sousa, Marcela Antonini, Daniel de Macedo Rocha, Henrique Ciabotti Elias, Renata Karina Reis

**Affiliations:** 1 Universidade de São Paulo, Escola de Enfermagem de Ribeirão Preto, PAHO/WHO Collaborating Centre for Nursing Research Development, Ribeirão Preto, SP, Brazil.; 2 Universidade Estadual do Maranhão, Curso de Enfermagem, Coroatá, MA, Brazil.; 3 Scholarship holder at the Coordenação de Aperfeiçoamento de Pessoal de Nível Superior (CAPES), Brazil.

**Keywords:** Validation Study, HIV, Educational Technology, Disease Prevention, Access to Information, Nursing

## Abstract

to build and validate an information portal on the combined prevention of human immunodeficiency virus infection.

a four-stage methodological study: definition, architecture, design, and implementation. The validation process was carried out by 24 nurses and 23 professionals specializing in Information Technology. A Likert-type questionnaire described the agreement between nurses/experts and IT professionals concerning the different attributes. The Content Validity Index was considered to have a cut-off point equal to or greater than 0.80.

the agreement rates were satisfactory, totaling 0.94 overall among nurses and 0.96 among IT professionals. The validity indicators showed satisfactory agreement in the attributes of general impression (0.96), objectives (0.90), adequacy of content (0.98) and language (1.00), relevance (0.96), high potential for attractiveness (0.91), and for innovation (0.90). In addition, the proposed technology has resources that guarantee easy navigation (0.96), as well as the quality of the interface (0.96), aesthetics, and audiovisuals (0.99).

it can be concluded that PREVI@IDST is an innovative educational resource because it brings together valid evidence for information and guidance on combined HIV prevention.

## Highlights

**(1)** The Information Portal is an innovative and valid resource. **(2)** It brings together valid evidence for information and guidance on HIV prevention. **(3)** The content on the portal is validated. **(4)** It is a new health education technology, especially for nursing. **(5)** It can be used in different services in the healthcare network.

## Introduction

Infection with the Human Immunodeficiency Virus (HIV) and AIDS is still understood as a global challenge, with an epidemiological, social, cultural, economic, and health impact that has been widely documented in the literature. It is a chronic, emerging, progressive, and universal condition, which, even in the face of important advances, the expansion and effectiveness of strategies for combined prevention, the introduction and free distribution of Antiretroviral Therapy (ART), shows significant notifications, both in low-, middle- and high-income countries^(1-2)^.

Estimated global data has shown the magnitude of the problem. In 2023, 39.9 million people were living with HIV; 1.3 million new cases were reported; 9.2 million were untreated, and 630,000 died from AIDS-related complications^(3)^.

In Brazil, although there is a downward trend in new infections for all ages and social segments, the greatest epidemiological impact is reported in key populations, groups disproportionately affected compared to the general population. In this perspective, the increased vulnerability of men who have sex with men, sex workers, injecting drug users, transgender people, or people deprived of their liberty, and the interference of socio-economic factors and health practices, as well as the level of information and knowledge, has been observed^(4-5)^.

The increased risk of HIV infection is also reported in other populations, such as adolescents, Indigenous people, pregnant women, black people, and people living on the streets, who should be prioritized due to the historical, educational, and structural contexts in which they live. These aspects can overlap, potentiate, and aggravate vulnerability factors, as well as cause substantial changes in the profile of people with HIV since the beginning of the epidemic^(6)^.

The conditions mentioned above reflect the presence of obstacles, barriers, and care challenges for tackling and controlling the HIV/AIDS epidemic in Brazil. They may explain the high prevalence and disproportionate burden of infection. They also limit the perception of vulnerability, are associated with the occurrence of high-risk practices, restrict access to specialized services for prevention and treatment, and contribute to late diagnosis, greater clinical impact, and maintenance of the transmission cascade^(7-8)^.

In light of this, the combined prevention approach to HIV stands out. This is an approach that integrates multiple prevention strategies to maximize the reduction of new infections. It combines biomedical, behavioral, and structural methods based on scientific evidence and respect for human rights, adapting to local realities. This approach offers a variety of prevention tools and options so that people can choose the ones that best suit their needs and preferences, to use them individually or in combination, such as the consistent use of condoms, the testing and treatment of Sexually Transmitted Infections (STIs) and the use of Pre-Exposure Prophylaxis (PrEP) for HIV. Combined prevention encompasses both primary prevention, aimed at people who are HIV-negative, and strategies to prevent transmission of the virus^(9-10)^.

Biomedical strategies are those that involve the use of medical technologies to prevent HIV infection, such as PrEP, Post-Exposure Prophylaxis (PEP), immunization for HBV and Human Papillomavirus (HPV), and treatment of STIs, among others. As for behavioral strategies, these involve regular HIV testing (for early identification of infection and adherence to therapy), as well as popular participation in sexual health education programs, to increase knowledge and information about the virus and its forms of prevention^(9,11)^.

Another recommended strategy is the incorporation of digital educational technologies in this context. Their effects have been explored and indicate a potential for disseminating and improving prevention messages, as well as positively impacting the level of knowledge, critical capacity, decision-making, and adherence to safe behaviors^(11-13)^. They are considered powerful and versatile tools that can be used to produce innovative strategies to improve STI/HIV prevention services^(13-14)^.

In nursing, digital educational health technologies are promising and attractive methods that guide care and educational practices by promoting greater adherence to continuous care, welcoming self-care, monitoring risk conditions, evaluating results, and expanding individual and collective knowledge, attitudes, and skills. However, there are still gaps in knowledge about the development of Information and Communication Technologies (ICT) with a focus on combined HIV prevention, as well as their effects on access to care, the level of knowledge, health behavior, and information of the population.

It is known that an effective response to reducing the incidence of HIV requires a knowledge-based policy, especially for the most vulnerable population, who have less access to health services^(16)^.

Given the scientific and healthcare advances that propose perspectives, proposals, and targets for controlling the epidemic around the world, it is essential to build and validate educational technologies in care before applying, disseminating, and incorporating them into clinical practice, especially those aimed at providing information on combined HIV prevention^(10-11)^.

Misinformation and low health knowledge are among the challenges to achieving the goals of controlling the HIV/AIDS epidemic by 2030^(9)^. Thus, promoting innovative solutions on combined HIV prevention with the potential to reach vulnerable populations is an urgent need. In this sense, this study aimed to build and validate an information portal on the combined prevention of HIV infection.

## Method

### Study design

This is a methodological study outlined in four stages of investigation: definition, architecture, design, and implementation^(17-18)^. This research meets the recommendations of the Standards for Quality Improvement Reporting Excellence (SQUIRE 2.0), as it is an approach to improving healthcare.

### Place of data collection

This study was carried out at a Higher Education Institution located in the municipality of Ribeirão Preto (SP), Brazil.

### Period

Data was collected between March and August 2022.

### Population

Health and IT experts took part in the development and validation stages of the educational portal on combined HIV prevention.

### Selection criteria

Inclusion was based on the criteria proposed by Fehring^(19)^, widely used in validation studies in different contexts and levels of health care^(17,20-22)^. Under these conditions, the criteria defined reflected the need for scientific, teaching, and/or care experience in preventing and dealing with the HIV epidemic and/or in the production of educational technologies. To this end, a panel of experts was drawn up. The selection was made after consulting the Lattes Platform of the National Council for Scientific and Technological Development (CNPq). In addition, researchers registered with the National Nursing Network on Sexually Transmitted Diseases/AIDS (RENAIDST) from all regions of Brazil were considered.

The judges from the Information Technology (IT) area were professionals with experience in the development of digital information technologies, software design, and programming. Recruitment took place via email from the IT department of the developing institution. No specific exclusion criteria were applied, and there were losses of participants who did not answer the questionnaires after three consecutive invitations and/or those who failed to complete the validation instruments.

### Participants

The systematized search identified 36 nurses with potential for inclusion. For the validation stage, 24 nurses with knowledge, clinical practice, and scientific production in the area of interest and 23 IT specialists took part and answered the questionnaire, constituting a convenience sample. However, the number of specialists included met the minimum as described in the literature^(23)^ and other studies^(24)^.

### Study variables

There aren’t any.

### Instruments used to collect information

The virtual platform Research Electronic Data Capture (REDCap^®^) was used, and an online questionnaire was applied to collect data, divided into two parts. The first part contained variables aimed at characterizing the sociodemographic, work, and educational background of the experts, and the second was made up of questions that measured agreement on the following attributes: general impression, objective, content, relevance, verbal language, attractiveness, innovation and the need to include topics. All the evaluation attributes were distributed on a Likert-type scale, with a four-point response range (strongly disagree to strongly agree).

For the IT judges, in addition to the characterization, a Likert-type questionnaire was used to assess the ergonomics and interface of the educational portal, as well as the layout, layout, screen format, buttons and navigation features, and aesthetic and audiovisual quality.

### Data collection

Data was collected in four stages.

### Stage 1: Definition

Stage 1 involved planning the educational technology, structuring the target audience, designing navigation resources, searching for and selecting content related to strategies for the combined prevention of HIV infection, as well as defining the objective and target audience, which included sexually active people regardless of HIV serology.

As part of this process, a bibliographic search was carried out using electronic databases, as well as manuals, guides, protocols, and recommendations proposed by national and international bodies, to provide an adequate reference on the magnitude of the problem, prevention strategies, and factors associated with greater vulnerability to transmission.

In addition, the content was prepared by a group of nurses and postgraduate researchers with experience in the subject of interest and reviewed by a researcher with technical, scientific, and technological expertise in the content. Given this, the quality assumptions for the preparation of educational materials were followed, which consider simple and accessible language, as well as the restriction of technical terms, as attributes favorable to the understanding of the content by the target audience^(25)^.

### Stage 2: Architecture

The second stage involved designing the interface, visual resources, logical organization, and hierarchical structuring of the content. This process included the systematic planning of the technology and aimed to define the layout of the consultation elements and the formal aesthetic order, as well as ensuring ease of communication and interaction with the user^(17)^. The aim was to propose a technological model that was easy to navigate and had the elements needed to guarantee accessibility for users.

### Stage 3: Design

The interface elements were integrated with the visual composition for the graphic-visual presentation of the portal. Consideration was given to presenting the aesthetics of the technology, defining the visual identity, symbols, colors, and typography, as well as the functionalities, navigation elements, animations, illustrations, infographics, forms, textual presentation, tables, links, label system, and titles. All the stages were conducted by the researchers and a communications and technology professional with extensive experience in producing health education materials.

### Stage 4: Implementation

An assessment of the information portal was carried out by judges/health and IT experts.

### Data processing and analysis

The data was analyzed descriptively to characterize the judges using the Statistic Package for the Social Sciences (SPSS) software, version 25.0. The CVI was adopted as a parameter for validity, with scores equal to or greater than 0.80 considered satisfactory. It was calculated by dividing the number of judges who rated the item as strongly agree/agree by the number of items, as described in other studies^(21,26)^. Global indicators (CVI-global) were also calculated to measure the judges’ agreement as to the representativeness of the attributes evaluated.

### Ethical aspects

The project was approved by the institution’s Research Ethics Committee (REC), process no. 5.068.52, on October 28, 2021. Participation was voluntary and conditional on signing the Free and Informed Consent Term (FICT).

## Results

**Figure 1 -:**
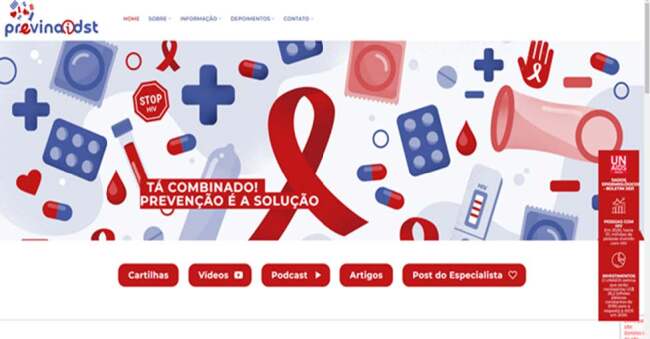
Graphic representation of the PREVIN@IDST portal. Ribeirão Preto, SP, Brazil, 2022

The information portal entitled PREVIN@IDST was built and validated (Figure 1). The information portal is an Internet-based website that hosts different content and media, such as booklets, videos, and podcasts, and allows researchers and users to create and communicate with each other. It also provides information on the care strategies developed by the Brazilian Testing and Counseling Centers (TCC) and PrEP services.

The portal can reach a large segment of the Brazilian population, given its free access via the link: https://gruposdepesquisa.eerp.usp.br/sites/previnaidst/. In addition, an attempt was made to use easy-to-understand language to approach the combined prevention of HIV infection.

To present the visual identity, the “@” symbol was used to characterize the computerized aspect, and the use of blue and red to represent the different methods of combined prevention. The research team designed the navigation layout after defining the access flow, usability methods, and attractiveness elements (Figure 1). All the resources used aimed to lead the user to an informative, interactive, dynamic, and up-to-date reading on combined HIV prevention.

**Figure 2 -:**
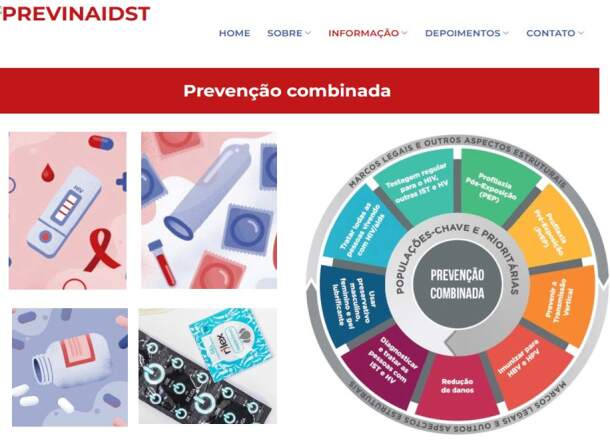
Infographics and media resources adopted for information on combined HIV prevention. Ribeirão Preto, SP, Brazil, 2022

The information content was structured based on an analysis of recommendations and clinical guidelines and encompassed the different strategies for the combined prevention of HIV infection (Figure 2), which include measures aimed at periodic testing, consistent condom use, treatment of other infections, prevention of vertical transmission, immunization, PrEP, and PEP adherence. It is worth highlighting the structuring of a specific guide that considers the dissemination of HIV treatment with viral load control as prevention.

**Figure 3 -:**
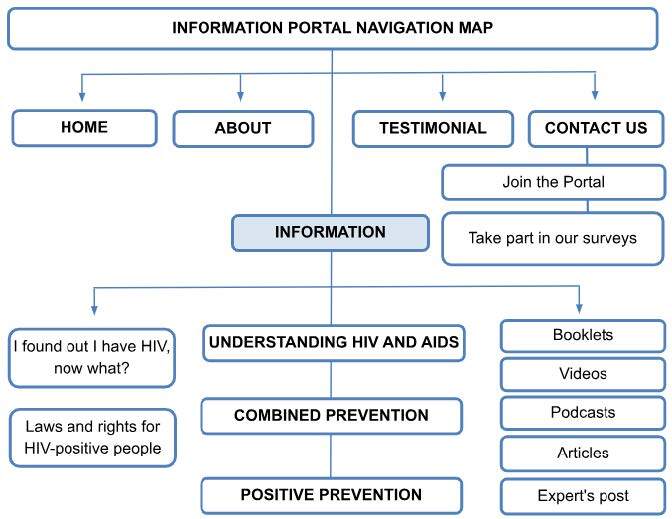
Navigation map of the information portal. Ribeirão Preto, SP, Brazil, 2022

The map, navigation structure, access flow, and usability elements are shown in Figure 3.

Once the portal had been built, implementation took place to validate it with the experts. For this stage, 24 nurses with knowledge, clinical experience, and scientific production in the area of interest and 23 IT specialists took part. Most nurses were female (18; 75%), with an average age of 39.3 (SD=10.3) years. Participants had experience in care (20; 83.3%) and teaching (6; 25.1%), especially in nursing education. The average time they had been working was 8.1 years (SD= 8.1) [0;30], and the frequency of participants with scientific production in their area of interest was significant (23; 95.8%).

As for the IT judges, there was a predominance of males (13; 56.5%), with an average age of 35.6 (SD=9.2) years. The evaluation of training and professional experience showed experience in different areas of design and web design (9; 38.7%) for an average time of 8.7 years. In addition, 17 (73.9%) participants had scientific production in the area.

## PREVIN@IDST agreement and validity indicators

In the evaluation of agreement by health specialists, the CVI for the attributes evaluated ranged from 0.90 to 1.0, and the total CVI was 0.94. The “general impression” attribute had a total CVI of 0.96, which indicated a satisfactory layout, stimulating reading, layout, themes, references, fonts, and adequate colors.

As for the proposed objectives (0.90), there was a high potential for informing people living with HIV and their sexual partners about combined prevention. Only the item related to PrEP-seeking locations achieved a CVI of 0.75, considering the geographical description of specialized services in different municipalities in the state of São Paulo.

In the evaluation of content (0.98), all the items evaluated achieved CVIs above 0.90. These indicators reflected the pertinence, usefulness, and appropriateness of the information for the target audience, as well as its potential use for health education processes and for promoting information, guidance, and combined HIV prevention.

The evaluated attribute “written language” (1.00) demonstrated clarity, objectivity, easy assimilation, and comprehension by the target audience. Other attributes evaluated were: “attractiveness” (0.91), “innovation” (0.90), and “inclusion of topics” (0.84). It is therefore considered that the use of audiovisual resources, the language adopted, and the colors used all contributed to the creation of an attractive, interesting, relevant, and pertinent technology.

The suggestions made were accepted and involved the need to explore combined prevention strategies through media resources. The targeting of measures at different genders and sexual orientations, such as women who have sex with women, was mentioned by the experts. The indicators of agreement and the attributes assessed by the health experts are described in Table 1.

**Table 1 -:** Indicators of agreement and attributes assessed by health experts (n = 24). Ribeirão Preto, SP, Brazil, 2022

**Attributes**	**I strongly agree**	**I agree**	**I disagree**	**I strongly disagree**	CVI*
	**n (%)**	**n (%)**	**n (%)**	**n (%)**	
**Overall impression**					**0.96**
Makes a good impression	17(70.8)	07(29.2)	-	-	1.00
Encourages reading	14(58.3)	09(37.5)	-	-	1.00
Satisfactory layout	12(50.0)	10(41.7)	01(4.2)	01(4.2)	0.92
The topics covered are appropriate	18(75.0)	06(25.0)	-	-	1.00
The colors are not distracting	15(62.5)	05(20.8)	03(12.5)	01(4.2)	0.83
The layout is conducive to understanding the message	15(62.5)	08(33.3)	01(4.2)	-	0.96
The font size is satisfactory for reading	14(58.3)	10(41.7)	-	-	1.00
The references are relevant	18(75.0)	06(25.0)	-	-	1.00
**Objectives**					**0.90**
Informs people living with HIV and their sexual partners about prevention	17(70.8)	06(25.0)	01(4.2)	-	0.96
Informs about combined HIV prevention	16(66.7)	08(33.3)	-	-	1.00
Represents the site search source for PrEP ^†^	11(45.8)	07(29.2)	05(20.8)	1(4.2)	0.75
**Contents**					**0.98**
The content facilitates the process of health education on the subject	20(83.3)	04(16.7)	-	-	1.00
The content enables the topic to be understood	15(62.5)	07(29.2)	01(4.2)	-	0.96
The content follows a logical sequence	19(79.2)	05(20.8)	-	-	1.0
The guidelines presented have been approached correctly	18(75.0)	06 (25.0)	-	-	1.0
The technical terms are properly defined	18(75.0)	04(16.7)	02(8.3)	-	0.92
The information is satisfactory and related to the intended purpose	18(75.0)	06(25.0)	-	-	1.0
There is no unnecessary information	18(75.0)	06(25.0)	-	-	1.0
The information is appropriate for the target audience	19(79.2)	04(16.7)	01(4.2)	-	0.96
The information is presented in a context relevant to the target audience	18(75.0)	05(20.8)	01(4.2)	-	0.96
Content is consistent with combined HIV prevention	20(83.3)	03(12.5)	01(4.2)	-	0.96
**Relevance**					**0.96**
The portal is relevant for informing the population about combined HIV prevention	22(91.7)	02(8.3)	**-**	**-**	1.00
The images and links represent important aspects for knowledge about combined HIV prevention	18(75.0)	05(20.8)	01(4.2)	**-**	0.96
Images, links, and photographs are relevant to understanding risky behavior and practices	16(66.7)	06(25.0)	02(8.3)	**-**	0.92
**Written language**					**1.0**
The written language used on the portal is accessible to the target audience	18(75.0)	6(25.0)	-	-	1.0
The verbal language is easy to understand	15(62.5)	9(37.5)	-	-	1.0
The concepts are clear and objective	16(66.7)	7(29.2)	-	-	1.0
**Attractiveness**					**0.91**
The visual composition is attractive and well-organized	12(50.0)	10(41.7)	01(4.2)	01(4.2)	0.92
Different audiovisual resources are used	12(50.0)	09(37.5)	03(12.5)	-	0.88
The different audiovisual resources are attractive for the portal	09(37.5)	10(41.7)	03(12.5)	01(4.2)	0.83
The language is attractive	15(62.5)	09(37.5)	-	-	1.00
The images are interesting	13(54.2)	09(37.5)	01(4.2)	01(4.2)	0.92
The images and links are integrated with the textual content	16(66.7)	07(29.2)	01(4.2)	-	0.96
The information is relevant to the target audience	19(79.2)	04(16.7)	01(4.2)	-	0.96
The portal makes a good impression	19(79.2)	05(20.8)	-	-	1.00
The portal encourages reading	17(70.8)	06(25.0)	01(4.2)	-	0.96
The colors used do not hinder reading	16(66.7)	05(20.8)	03(12.5)	-	0.88
The font size and format are satisfactory for reading	15(62.5)	06(25.0)	03(12.5)	-	0.88
**Innovation**					**0.90**
It has a unique design	11(45.8)	10(41.7)	03(12.5)	-	0.88
Represents the innovative idea	11(45.8)	11(45.8)	02(8.3)	-	0.92
It is a different way of disseminating information	13(54.2)	10(41.7)	01(4.2)	-	0.96
It’s relaxed	11(45.8)	12(50.0)	01(4.2)	-	0.96
Presents a language that brings it closer to the public	14(58.3)	10(41.7)	-	-	1.0
Addresses information for different sexual orientations	16(66.7)	05(20.8)	02(8.3)	-	0.91
Addresses information for different genders	14(58.3)	05(20.8)	05(20.8)	-	0.79
Communicates with commonly used social networks	12(50.0)	09(37.5)	02(8.3)	01(4.2)	0.88
It is unprecedented with content on combined HIV prevention	11(45.8)	08(33.3)	05(20.8)	-	0.79
**IVC-Global** ^‡^					**0.94**

*CVI = Content Validity Index; ^†^PrEP = Pre-Exposure Prophylaxis; ^‡^CVI-Global = Global Content Validity Index

In the evaluation by the judges from the IT area, the portal had an overall CVI of 0.97 (Table 2). The evaluation also showed interface quality (0.96), aesthetic and audiovisual quality (0.99), and ease of navigation (0.96). All the items were satisfactory, according to the benchmark adopted, which indicated evidence of validity in indices equal to or greater than 0.80. This result shows that the portal has adequate indicators of aesthetic and audiovisual quality and that the format, interface, and structured layout guarantee ease and safety for navigation, as shown in Table 2.

**Table 2 -:** Indicators of agreement and the attributes evaluated by information technology specialists (n = 23). Ribeirão Preto, SP, Brazil, 2022

**Attributes**	**I strongly agree**	**I agree**	**I disagree**	**I strongly disagree**	CVI*
	**n (%)**	**n (%)**	**n (%)**	**n (%)**	
**Interface quality**					**0.96**
Satisfactory layout	16(69.6)	07(30.4)	-	-	1.00
The visual aspect is good	16(69.6)	07(30.4)	-	-	1.00
The format of the screens is satisfactory	13(56.6)	10(43.4)	-	-	1.00
The navigation buttons are satisfactory	15(65.2)	07(30.4)	01(4.4)	-	0.96
The colors used do not hinder reading	10(43.4)	10(43.4)	02(8.8)	01(4.4)	0.87
The layout is conducive to understanding the message	10(43.4)	12(52.2)	01(4.4)	-	0.96
The font chosen is legible	12(52.1)	09(39.1)	02(8.8)	-	0.91
The size of the font chosen is good	08(34.8)	15(65.2)	-	-	1.00
**Aesthetic and audiovisual quality**					**0.99**
The quality and resolution of the photographs are satisfactory	13(56.6)	10(43.4)	-	-	1.00
The quality of the images (infographics) is satisfactory	11(50.0)	11(50.0)	-	-	1.00
The quality of the links is satisfactory	17(73.9)	06(26.1)	-	-	1.00
The quality of the texts is satisfactory	13(56.6)	09(39.1)	01(4.3)	-	0.96
**Easy navigation**					**0.96**
Information is easily found within the portal	12(52.2)	11(47.8)	-	-	1.00
Information about the portal can be found within its own platform	09(39.1)	14(60.9)	-	-	1.00
Information on security and privacy can be found within the portal	12(52.2)	11(47.8)	-	-	1.00
Information on security and privacy is easy to find within the portal	07(30.5)	14(60.9)	01(4.3)	01(4.3)	0.91
You can easily contact the people responsible for the portal	10(43.4)	12(52,2)	01(4.4)	-	0.96
Pages load quickly	10(43.4)	12(52.1)	01(4.4)	-	0.96
There are not too many pop-ups (spontaneously appearing floating pages) within the portal	14(60.9)	08(34.8)	01(4.4)	-	0.96
No difficulty in accessing links	13(56.6)	08(34.7)	02(8.7)	-	0.91
There are direct and functional links to the portal’s social networks	10(43.4)	12(52.1)	-	01(4.4)	0.96
**IVC-Global** ^†^					**0.96**

*CVI = Content Validity Index; ^†^CVI-Global = Global Content Validity Index

## Discussion

The PREVIN@IDST portal has shown adequate validity among health and IT specialists. Digital educational technology can be a contemporary, didactic, interactive, and essential tool for providing information and popularizing strategies for combined HIV prevention. It also has great potential to contribute to achieving the epidemiological control targets proposed by the Joint United Nations Program on HIV/AIDS (UNAIDS).

The computerization of prevention is widely referred to in different care contexts. It is considered that no single resource will be enough to control the epidemic and that strategies, when combined, adapted to the profile of the population, and incorporated into educational technology, can increase access to information. Similarly, this perspective was pointed out in a study aimed at developing a serious game about safe sex and contraception for teenagers. The technological proposal, as well as representing an opportunity to approach the subject in an attractive, interactive way that is contextualized to the reality of the target audience, presented the potential to promote changes in behavior and contribute to information, education, and awareness among users^(27)^.

The information portal is an educational technology that has become popular due to its wide availability, ease of use, and practicality. The development of health technologies for the screening, monitoring, prevention, diagnosis, and treatment of STIs can enhance the quality of care indicators, the process, and the clinical outcome^(28)^. In the field of HIV prevention and treatment, they can provide users with the possibility of generating, sharing, interacting, and receiving information, with guaranteed anonymity and confidentiality of information^(29)^.

The high potential for disseminating information and reaching vulnerable populations are also attributes associated with incorporating educational technologies into health practices. The literature shows a relationship between the level of knowledge and significant changes in risk behaviors, as well as increased access to reception and testing services and adherence to prophylaxis and prevention strategies^(28,30-31)^.

Given that the effectiveness of HIV prevention strategies can vary, determining the sufficient level of evidence for their inclusion in the technology was a critical step. From this perspective, the structuring of the content was expressed by the strategic and simultaneous combination of biomedical, behavioral, structural, social, and risk management interventions that operate on multiple levels.

The different populations in vulnerable situations are the target audience for the educational actions proposed by PREVIN@IDST. This perspective is demonstrated by the combination of information strategies aimed both at achieving an undetectable viral load and at preventing the sexual transmission of HIV. Despite this, there are still groups of people living with HIV who remain at risk of transmission to their sexual partners. Thus, the population diversity considered by the technology shows that HIV prevention will be more effective when the different points in the transmission cycle are prevented.

Around the world, public policies, government campaigns, and technologies for HIV prevention are strongly aimed at people who are HIV-negative and vulnerable to infection. However, this segment comprises a large and diverse group to be targeted with high coverage. In this sense, epidemiological projections indicate that, in 30 years, few countries have managed to reverse the HIV epidemic based on strategies aimed at seronegative people^(32)^.

Thus, optimizing HIV prevention must also consider the high proportion of people living with HIV/AIDS. In this way, the potential components of combined HIV prevention, as well as focusing on interventions with proven efficacy or promising indications for reducing susceptibility (consistent condom use, behavioral risk reduction, and pre- and post-exposure prophylaxis), incorporated testing and adherence to ART, the effective therapeutic resource for controlling and maintaining viral load at undetectable levels, as well as for delaying the progression of the disease and preventing transmission of the infection^(8)^.

The portal also addresses the need for regular HIV testing. Expanding access to knowledge of HIV status is a global priority, both to link positive cases to clinical care and to prevent new infections. Lack of knowledge about the possibilities, timing, and indication of HIV testing, as well as one’s own serological status, are barriers to controlling the epidemic and contribute to the occurrence of risky practices, which should be the target of popular information and awareness^(32)^.

The evaluation of the portal by specialists in the health area helped to adapt the content, objectives, and language to the epidemiological context, as well as the barriers and challenges experienced in Brazil and the characteristics of the target population. The participation of nurses, in particular, in this process was fundamental due to their leading role, knowledge, skills, and abilities for guidance, welcoming, information, and risk management in health-disease conditions, problems, or situations prevalent on the national scene^(21,33)^.

Validating educational materials in the information age allows for the structuring and availability of evidence-based content^(22)^. It also promotes clarity, objectivity, and relevance, as well as ensuring attractiveness by incorporating language and media resources that are simple, accessible, and easy to assimilate. Similar evidence is found in other studies that have evaluated the language of health education materials and found that the use of technical language is one of the main barriers to accessing information^(34-35)^.

PREVIN@IDST has the potential for innovation and popular information. In the literature, different websites on combined prevention have been developed. However, most of them focus on disseminating guidelines, protocols, and clinical recommendations for health professionals. The approach to population information is still limited, incipient, and little explored^(21-22)^.

The innovative nature of the portal also refers to the quality of the interface, both aesthetic and audiovisual, as well as the structuring of an interface for interaction and communication between researchers and users to create, make available, and exchange content in different media formats (booklets, podcasts, e-books, and educational videos).

It should be noted that the suggestions and criticisms made during the experts’ evaluation helped to redirect the technological resource towards the proposed objectives and made it possible to minimize potential biases on the part of the researchers in the development and validation process.

As a limitation of this study, it should be noted that the results of this production showed that the technological validity attributes were evaluated exclusively by nurses. Despite this, we highlight the potential of this category to intervene in health conditions prevalent in the population, as well as their experience in proposing multi-professional strategies to ensure popular education as a basis for health promotion and disease prevention. Future studies are suggested to measure its effects on the population’s health knowledge, attitude, and behavior.

The implications of this study for nursing and the promotion of sexual health refer to the availability of an attractive and free technology based on evidence of validity for informing different populations and vulnerable segments about combined HIV prevention. It also highlights its promising potential for use in educational and risk management interventions, as well as for the implementation of public policies that consider information and knowledge of the disease as necessary goals for controlling the epidemic.

## Conclusion

PREVI@IDST is an innovative and valid educational resource, as it brings together valid evidence for information and guidance on combined HIV prevention. The validity indicators highlighted the appropriateness of the content, objectives, and language, as well as the overall good impression, ease of navigation, relevance, innovation, attractiveness, aesthetic and audiovisual quality. In addition, the potential for information and health education for the different levels of care and population segments stands out.

## References

[1] Kemnic T. R., Gulick P. G. (2024). StatPearls [Internet]. Treasure Island.

[2] Chun H. M., Dirlikov E., Cox M. H., Sherlock M. W., Obeng-Aduasare Y., Sato K. (2023). Vital Signs: Progress Toward Eliminating HIV as a Global Public Health Threat Through Scale-Up of Antiretroviral Therapy and Health System Strengthening Supported by the U.S. President’s Emergency Plan for AIDS Relief - Worldwide, 2004-2022. MMWR Morb Mortal Wkly Rep.

[3] Joint United Nations Programme on HIV/Aids. (2024). https://unaids.org.br/estatisticas/.

[4] Mody A., Sohn A. H., Iwuji C., Tan R. K. J., Venter F., Geng E. H. (2024). HIV epidemiology, prevention, treatment, and implementation strategies for public health. Lancet.

[5] Brandelli C. A., Moura J. B., Silva J. M., Beloqui J. A., Espindola Y., Araujo C. F. (2022). Key and general population HIV-related stigma and discrimination in HIV-specific health care settings: results from the Stigma Index Brazil. AIDS Care.

[6] Ministério da Saúde (BR), Secretaria de Vigilância em Saúde. (2022). Boletim Epidemiológico - HIV/Aids 2022 [Internet].

[7] Toledo L. S. G., Palmieri P., Ribeiro G. R., Silva A., Bastos F. I. (2024). Barriers and facilitators for HIV rapid testing among transgender women and gay and other men who have sex with men in Brazil: A scoping review. Glob Public Health.

[8] Pereira C. R., Cruz M. M., Cota V. L., Almeida B. M. M. (2022). Linkage strategy and vulnerabilities in the barriers to HIV/AIDS treatment for men who have sex with men. Cien Saude Colet.

[9] Cambiano V., Miners A., Lampe F. C., McCormack S., Gill O. N., Hart G. (2023). The effect of combination prevention strategies on HIV incidence among gay and bisexual men who have sex with men in the UK: a model-based analysis. Lancet HIV.

[10] Kremer C., Kamali A., Kuteesa M., Seeley J., Hens N., Nsubuga R. N. (2023). Modelling the impact of combining HIV prevention interventions on HIV dynamics in fishing communities in Uganda. BMC Infect Dis.

[11] Gonçalves T. R., Costa A. H. C., Sales M. S., Leite H. M. (2020). Combined HIV prevention? Systematic review of interventions with women from low- and middle-income countries. Cien Saude Colet.

[12] Romero R. A., Klausner J. D., Marsch L. A., Young S. D. (2021). Technology-Delivered Intervention Strategies to Bolster HIV Testing. Curr HIV/AIDS Rep.

[13] Veronese V., Ryan K. E., Hughes C., Lim M. S., Pedrana A., Stoové M. (2020). Using Digital Communication Technology to Increase HIV Testing Among Men Who Have Sex With Men and Transgender Women: Systematic Review and Meta-Analysis. J Med Internet Res.

[14] Swendeman D., Rotheram-Borus M. J., Arnold E. M., Fernández M. I., Comulada W. S., Lee S. J. (2024). Optimal strategies to improve uptake of and adherence to HIV prevention among young people at risk for HIV acquisition in the USA (ATN 149): a randomised, controlled, factorial trial. Lancet Digit Health.

[15] Cao B., Bao H., Oppong E., Feng S., Smith K. M., Tucjer J. D. (2020). Digital health for sexually transmitted infection and HIV services: a global scoping review. Curr Opin Infect Dis.

[16] Tiittala P., Kivelä P., Liitsola K., Ollgren J., Pasanen S., Vasankari T. (2018). Important Gaps in HIV Knowledge, Attitudes and Practices Among Young Asylum Seekers in Comparison to the General Population. J Immigr Minor Health.

[17] Lima J. S. (2014). https://tede2.uefs.br:8080/handle/tede/90.

[18] Lins T. H., Marin H. F. (2012). Evaluation of a website on nursing care in the post anesthesia recovery room. Acta Paul Enferm.

[19] Fehring R. J., Carroll-Johnson R. M. (1994). The Fehring model.

[20] Melo E. S. (2019). Construção e validação de material educativo digital para redução do risco cardiovascular em pessoas vivendo com HIV [dissertation]. Escola de Enfermagem de Ribeirão Preto, Universidade de São Paulo.

[21] Cintra M. M. (2020). Desenvolvimento, validação, análise da acessibilidade e certificação internacional de um portal de informações sobre saúde e inclusão [thesis]. Escola de Enfermagem de Ribeirão Preto, Universidade de São Paulo.

[22] Lima I. D. A., CGRMP Leon, Ribeiro L. M., Silva I. C. R., Vilela D. M., Fonseca L. M. M. (2022). A Serious Game (Immunitates) About Immunization: Development and Validation Study. JMIR Serious Games.

[23] Pasquali L. (2009). Instrumentação psicológica: fundamentos e práticas.

[24] Soares I. A. A., Góes F. G. B., Silva A. C. S. S., Pereira-Ávila F. M. V., Oliveira G. B., Silva M. A. (2024). Health education website on home care for newborns: construction, validation, and evaluation. Rev. Latino-Am. Enfermagem.

[25] Daher J., Vijh R., Linthwaite B., Dave S., Kim  J., Dheda K. (2017). Do digital innovations for HIV and sexually transmitted infections work? Results from a systematic review (1996-2017). BMJ Open.

[26] Jesus G. J., Caliari J. S., Oliveira L. B., AAFLN Queiroz, Figueiredo R. M., Reis R. K. (2020). Construction and validation of educational material for the health promotion of individuals with HIV. Rev. Latino-Am. Enfermagem.

[27] Tamashiro L. M. C., Fonseca L. M. M. (2024). Development of a serious game for learning about safe sex and contraception in adolescence. Rev. Latino-Am. Enfermagem.

[28] Schnall R., Kuhns L. M., Pearson C., Batey D. S., Bruce J., Hidalgo M. A. (2022). Efficacy of MyPEEPS Mobile, an HIV Prevention Intervention Using Mobile Technology, on Reducing Sexual Risk Among Same-Sex Attracted Adolescent Males: A Randomized Clinical Trial. JAMA Netw Open.

[29] Shrestha R., Maviglia F., Altice F. L., DiDomizio E., Khati A., Mistler C. (2022). Mobile Health Technology Use and the Acceptability of an mHealth Platform for HIV Prevention Among Men Who Have Sex With Men in Malaysia: Cross-sectional Respondent-Driven Sampling Survey. J Med Internet Res.

[30] Mehraeen E., SeyedAlinaghi S., Pashaei Z., Mirzapour P., Barzegary A., Vahedi F. (2022). Mobile applications in HIV self-management: A systematic review of scientific literature. AIDS Rev.

[31] Blažić T. N., Bogdanić N., Nola I. A., Ličina M. L. K., Aždajić M. D. (2022). Digital technology and HIV, HCV and STI voluntary counselling and testing: good practice example from Croatia. Cent Eur J Public Health.

[32] Kurth A. E., Celum C., Baeten J. M., Vermund S. H., Wasserheit J. N. (2011). Combination HIV prevention: significance, challenges, and opportunities. Curr HIV/AIDS Rep.

[33] Relf M. V. (2022). Nurses, Nurse-Led Interventions, and Nursing Models of Care: Essential in HIV Prevention, Care, and Treatment. J Assoc Nurses AIDS Care.

[34] Melo E. S., Antonini M., Costa C. R. B., Pontes P. S., Gir E., Reis R. K. (2022). Validation of an interactive electronic book for cardiovascular risk reduction in people living with HIV. Rev. Latino-Am. Enfermagem.

[35] Arifin B., Rokhman M. R., Zulkarnain Z., Perwitasari D. A., Manggau M., Rauf S. (2022). Adaptation and validation of the HIV Knowledge Questionnaire-18 for the general population of Indonesia. Health Qual Life Outcomes.

