# Supplementary material for: Construction and validation of an information portal on combined HIV prevention
Source: Rev Lat Am Enfermagem. 2025 May 2;33:e4509. doi: 10.1590/1518-8345.7221.4509 (PMC12052314; doi:10.1590/1518-8345.7221.4509)
Supplement: Translation

# Translation: Construção e validação de Portal Informativo sobre a prevenção combinada do HIV*

**DOI:** 10.1590/1518-8345.7221.4510

**Authors:** Felipe Martins Lioi, Laelson Rochelle Milanês Sousa, Marcela Antonini, Daniel de Macedo Rocha, Henrique Ciabotti Elias, Renata Karina Reis

**Affiliations:** 1 Universidade de São Paulo, Escola de Enfermagem de Ribeirão Preto, Centro Colaborador da OPAS/OMS para o Desenvolvimento da Pesquisa em Enfermagem, Ribeirão Preto, SP, Brasil.; 2 Universidade Estadual do Maranhão, Curso de Enfermagem, Coroatá, MA, Brasil.; 3 Bolsista da Coordenação de Aperfeiçoamento de Pessoal de Nível Superior (CAPES), Brasil.

**Keywords:** Estudo de Validação, HIV, Tecnologia Educacional, Prevenção de Doenças, Acesso à Informação, Enfermagem

## Abstract

construir e validar um portal de informações sobre a prevenção combinada da infecção pelo vírus da imunodeficiência humana.

estudo metodológico delineado em quatro etapas: definição, arquitetura, *design* e implementação. O processo de validação foi realizado por 24 enfermeiros e 23 profissionais especialistas em Tecnologia da Informação. Foi aplicado um questionário tipo Likert, para descrever a concordância entre enfermeiros/*experts* e profissionais da área de Tecnologia da Informação em relação aos diferentes atributos. Considerou-se o Índice de Validade de Conteúdo com ponto de corte igual ou superior a 0,80.

os índices de concordância foram satisfatórios, totalizando 0,94 global entre os enfermeiros e 0,96 entre os profissionais de Tecnologia da Informação. Os indicadores de validade demonstraram satisfatória concordância nos atributos impressão geral (0,96), objetivos (0,90), adequação do conteúdo (0,98) e da linguagem (1,00), relevância (0,96), alto potencial para atratividade (0,91) e para inovação (0,90). Ainda, a tecnologia proposta apresenta recursos que garantem facilidades para a navegação (0,96), assim como a qualidade da interface (0,96), da estética e do audiovisual (0,99).

conclui-se que o PREVIN@IDST configura-se como um recurso educacional inovador por reunir as evidências válidas para a informação e a orientação sobre a prevenção combinada do HIV.

## Destaques

**(1)** O Portal Informativo é um recurso inovador e válido. **(2)** Reúne evidências válidas para informação e orientação sobre a prevenção do HIV. **(3)** O conteúdo no portal está validado. **(4)** Trata-se de uma nova tecnologia de educação em saúde, sobretudo para a Enfermagem. **(5)** Pode ser utilizada em diferentes serviços da rede de atenção à saúde.

## Introdução

A infecção pelo Vírus da Imunodeficiência Humana (HIV) e pela aids ainda é compreendida como um desafio global, com um impacto epidemiológico, social, cultural, econômico e de saúde amplamente documentado na literatura. Trata-se de uma condição crônica, emergente, progressiva e universal, que mesmo diante dos importantes avanços, da ampliação e da efetividade das estratégias para a prevenção combinada, introdução e distribuição gratuita da Terapia Antirretroviral (TARV), apresenta notificações expressivas, tanto em países de baixa, quanto de média e alta renda^(1-2)^.

Os dados globais estimados demonstraram a magnitude do problema. Em 2023, 39,9 milhões de pessoas viviam com HIV; 1,3 milhão de casos novos foram notificados; 9,2 milhões estavam sem tratamento e 630 mil morreram por complicações relacionadas à aids^(3)^.

No Brasil, embora seja evidenciada a tendência decrescente nas novas infecções para todas as idades e segmentos sociais, o maior impacto epidemiológico é reportado em populações-chave, grupos afetados de forma desproporcional, quando comparados à população geral. Nessa perspectiva, a vulnerabilidade é aumentada em homens que fazem sexo com homens, trabalhadores do sexo, usuários de drogas injetáveis, pessoas transgênero ou privadas de liberdade e por interferência dos fatores socioeconômicos e das práticas em saúde, assim como pelo nível de informação e conhecimento^(4-5)^.

O risco aumentado para a infecção pelo HIV também é referenciado em outras populações, como: adolescentes, indígenas, gestantes, pessoas negras ou em situação de rua, que devem ser priorizadas em virtude dos contextos históricos, educacionais e estruturais nos quais estão inseridos. Estes aspectos podem sobrepor, potencializar e agravar os fatores de vulnerabilidade, além de provocar mudanças substanciais no perfil das pessoas com HIV desde o início da epidemia^(6)^.

As condições supracitadas refletem a presença de obstáculos, barreiras e desafios assistenciais para o enfrentamento e o controle da epidemia do HIV/aids no Brasil, e podem explicar a alta prevalência e a carga desproporcional da infecção. Ainda, limitam a percepção de vulnerabilidade, associam-se à ocorrência de práticas de alto risco, limitam o acesso aos serviços especializados para a prevenção e o tratamento, contribuem para o diagnóstico tardio, para o maior impacto clínico e a manutenção da cascata de transmissão^(7-8)^.

Diante disso, destaca-se a abordagem da prevenção combinada ao HIV. Trata-se de uma abordagem que integra as múltiplas estratégias de prevenção para maximizar a redução de novas infecções. Ela combina métodos biomédicos, comportamentais e estruturais, baseados em evidências científicas e no respeito aos direitos humanos, adaptando-se às realidades locais. Essa abordagem oferece uma variedade de ferramentas e opções de prevenção para que as pessoas possam escolher as que melhor se adequam às necessidades e preferências, a fim de utilizá-las de forma individual ou em combinação, como por exemplo o uso consistente de preservativos, a testagem e o tratamento de Infecções Sexualmente Transmissíveis (IST) e o uso de Profilaxia Pré-exposição (PrEP) para o HIV. A prevenção combinada abrange tanto a prevenção primária, destinada às pessoas com sorologia negativa para o HIV, quanto as estratégias para evitar a transmissão do vírus^(9-10)^.

As estratégias biomédicas são aquelas que envolvem o uso de tecnologias médicas para prevenir a infecção pelo HIV, como PrEP, Profilaxia Pós-Exposição (PEP), imunização para HBV e Papilomavírus Humano (HPV), tratamento das IST, dentre outras. Quanto às estratégias comportamentais, estas envolvem a testagem regular para o HIV (para a identificação da infecção e a adesão terapêutica precoces), assim como a participação popular em programas de educação sobre a saúde sexual, a fim de aumentar o conhecimento e a informação sobre o vírus e as suas formas de prevenção^(9,11)^.

Outra estratégia recomendada é a incorporação de tecnologias educacionais digitais nesse contexto. Os seus efeitos são explorados e indicam potenciais para difundir e melhorar as mensagens de prevenção, além de impactar positivamente o nível de conhecimento, a capacidade crítica, o processo de tomada de decisão e a adesão aos comportamentos seguros^(11-13)^. São consideradas ferramentas poderosas e versáteis que podem ser utilizadas na produção de estratégias inovadoras para melhorar os serviços de prevenção das IST/HIV^(13-14)^.

Na Enfermagem, as tecnologias educacionais digitais em saúde configuram-se como métodos promissores e atraentes que direcionam as práticas assistenciais e educativas, por promover uma maior adesão ao cuidado contínuo, ao acolhimento, ao autocuidado, ao monitoramento de condições de risco, à avaliação de resultados e à ampliação de conhecimentos, atitudes e habilidades individuais e coletivas^(12,15)^. Entretanto, ainda há lacunas no conhecimento sobre o desenvolvimento de Tecnologias de Comunicação e Informação (TIC) com foco na prevenção combinada do HIV, assim como sobre os seus efeitos no acesso ao cuidado, no nível de conhecimento, no comportamento em saúde e na informação da população.

Sabe-se que uma resposta efetiva à redução da incidência de HIV requer uma política baseada em conhecimento, especialmente para a população mais vulnerável, que possui menor acesso aos serviços de saúde^(16)^.

Diante dos avanços científicos e assistenciais que propõem perspectivas, propostas e metas para o controle da epidemia no mundo, torna-se fundamental a construção e a validação de tecnologias educacionais no cuidado antes da sua aplicação, divulgação e incorporação na prática clínica, sobretudo daquelas direcionadas à informação sobre a prevenção combinada do HIV^(10-11)^.

A desinformação e o baixo conhecimento em saúde compreendem os desafios para atingir as metas de controle da epidemia da infecção pelo HIV/aids até 2030^(9)^. Assim, promover as soluções inovadoras sobre a prevenção combinada do HIV com potencial de alcançar as populações vulnerabilizadas constitui uma necessidade urgente. Nesse sentido, este estudo objetivou construir e validar um portal de informações sobre a prevenção combinada da infecção pelo HIV.

## Método

### Delineamento do estudo

Trata-se de um estudo metodológico delineado em quatro etapas de investigação: definição, arquitetura, *design* e implementação^(17-18)^. Esta pesquisa atende às recomendações do *Standards for Quality Improvement Reporting Excellence* (SQUIRE 2.0), por ser uma abordagem para a melhoria da assistência à saúde.

### Local da coleta de dados

Este estudo foi desenvolvido em uma Instituição de Ensino Superior localizada no município de Ribeirão Preto (SP), Brasil.

### Período

A coleta de dados ocorreu entre os meses de março e agosto de 2022.

### População

Especialistas em saúde e informática participaram das etapas de elaboração e validação do portal educacional sobre a prevenção combinada do HIV.

### Critérios de seleção

A inclusão foi condicionada aos critérios propostos por Fehring^(19)^, amplamente utilizados em estudos de validação em diferentes contextos e níveis de atenção à saúde^(17,20-22)^. Nessas condições, os critérios delimitados refletiram a necessidade de experiência científica, docente e/ou assistencial na prevenção e no enfrentamento da epidemia do HIV, e/ou na produção de tecnologias educacionais. Para tal, foi elaborado um painel de especialistas. A seleção ocorreu após a consulta à Plataforma Lattes do Conselho Nacional de Desenvolvimento Científico e Tecnológico (CNPq). Ademais, foram considerados os pesquisadores cadastrados na Rede de Enfermagem Nacional em Doenças Sexualmente Transmissíveis/AIDS (RENAIDST) procedentes de todas as regiões do Brasil.

Em relação aos juízes da área de Tecnologia da Informação (TI), participaram os profissionais com experiência no desenvolvimento de tecnologias digitais da informação, *design* e programação de *softwares*. O recrutamento ocorreu via *e-mail* da seção de TI da instituição desenvolvedora. Não foram aplicados critérios de exclusão específicos, e houve perdas de participantes que não responderam os questionários após três convites consecutivos e/ou que não completaram o preenchimento dos instrumentos de validação.

### Participantes

A busca sistematizada identificou 36 enfermeiros com potencial para a inclusão. Para a etapa de validação participaram 24 enfermeiros com conhecimento, atuação clínica e produção científica na área de interesse e 23 especialistas em TI, que responderam ao questionário, constituindo-se em amostra de conveniência. Porém, o número de especialistas incluídos atendeu ao mínimo conforme descrito na literatura^(23)^ e em outros estudos^(24)^.

### Variáveis do estudo

Não há.

### Instrumentos utilizados para a coleta das informações

Foi utilizada a plataforma virtual *Research Electronic Data Capture* (REDCap^®^) e aplicado um questionário *online* para a coleta de dados, dividido em duas partes. A primeira parte continha variáveis direcionadas para a caracterização sociodemográfica, laboral e educacional dos especialistas, e a segunda foi constituída por questões que permitiram mensurar a concordância nos seguintes atributos: impressão geral, objetivo, conteúdo, relevância, linguagem verbal, atratividade, inovação e necessidade de inclusão de tópicos. Todos os atributos de avaliação foram distribuídos em escala do tipo Likert, com variação de respostas em quatro pontos (discordo fortemente a concordo fortemente).

Para os juízes da área de TI, além da caracterização, foi aplicado um questionário do tipo Likert, com vistas a avaliar a ergonomia e a interface do portal educacional, bem como da estruturação do *layout*, diagramação, formato de telas, botões e recursos de navegação, qualidade estética e audiovisual.

### Coleta de dados

A coleta de dados foi realizada em quatro etapas.

### Etapa 1: definição

Na etapa 1 foram realizadas as atividades de planejamento da tecnologia educacional, assim como a estruturação do público-alvo, a elaboração dos recursos de navegação, a busca e a seleção do conteúdo relacionado às estratégias para a prevenção combinada da infecção pelo HIV, bem como pela definição do objetivo e do público-alvo, que compreendeu pessoas sexualmente ativas independente da sorologia para o HIV.

Nesse processo, foi realizada uma busca bibliográfica por meio da consulta eletrônica em bases de dados, assim como nos manuais, guias, protocolos e recomendações propostos por órgãos nacionais e internacionais, com o objetivo de fornecer um referencial adequado sobre a magnitude do problema, as estratégias de prevenção e os fatores associados a maior vulnerabilidade de transmissão.

Ainda, o conteúdo foi elaborado por um grupo de enfermeiros e pós-graduandos pesquisadores com experiência na temática de interesse e revisado por uma pesquisadora com *expertise* técnica, científica e tecnológica no conteúdo. Diante disso, seguiram-se os pressupostos de qualidade para a elaboração de materiais educativos que consideram a linguagem simples e acessível, assim como a restrição de termos técnicos, como os atributos favoráveis à compreensão do conteúdo pelo público-alvo^(25)^.

### Etapa 2: arquitetura

A segunda etapa foi relacionada à elaboração da interface, recursos visuais, organização lógica e estruturação hierárquica do conteúdo. Este processo compreendeu o planejamento sistematizado da tecnologia e visou definir a disposição dos elementos de consulta e a ordenação estético-formal, assim como garantir a facilidade para a comunicação e a interação com a pessoa usuária^(17)^. Buscou-se propor um modelo tecnológico de fácil navegação e com os elementos favoráveis à garantia da acessibilidade pelos usuários.

### Etapa 3: ***design***

Realizou-se a integração dos elementos de interface com a composição visual para a apresentação gráfico-visual do portal. Considerou-se a apresentação da estética da tecnologia, a definição da identidade visual, símbolos, cores e tipografia, além das funcionalidades, elementos de navegação, animações, ilustrações, infográficos, formulários, apresentação textual, tabelas, *links*, sistema de rótulos e de títulos. Todas as etapas foram conduzidas pelos pesquisadores e por profissional da comunicação e tecnologia, com ampla experiência na produção de materiais para educação em saúde.

### Etapa 4: implementação

Realizou-se a avaliação do portal de informações por juízes/especialistas da área da saúde e da TI.

### Tratamento e análise dos dados

Os dados foram analisados de forma descritiva para a caracterização dos juízes por meio do *software Statistic Package for the Social Sciences* (SPSS), versão 25.0. O IVC foi adotado como parâmetro para a validade, com os escores iguais ou superiores a 0,80 considerados satisfatórios. O cálculo ocorreu por meio da divisão do número de juízes que avaliaram o item como concordo fortemente/concordo pelo número de itens, conforme descrito em outros estudos^(21,26)^. Também foram calculados os indicadores globais (IVC-global) para mensurar a concordância dos juízes quanto à representatividade em relação aos atributos avaliados.

### Aspectos éticos

O projeto foi aprovado pelo Comitê de Ética em Pesquisa (CEP) da instituição, processo nº 5.068.52, em 28/10/2021. A participação foi voluntária e condicionada à assinatura do Termo de Consentimento Livre e Esclarecido (TCLE).

## Resultados

Construiu-se e validou-se o portal de informação intitulado PREVIN@IDST (Figura 1). O portal de informação é um *website* baseado na *Internet* que hospeda os diferentes conteúdos e as mídias, como as cartilhas, os vídeos, o *podcast*, e que permite a criação e a comunicação entre os pesquisadores e os usuários. Ainda, dispõe sobre as estratégias de cuidados desenvolvidas pelos serviços dos Centros de Testagem e Aconselhamento (CTA) e da PrEP brasileiros.

O portal pode alcançar um grande segmento da população brasileira, haja vista o seu acesso gratuito por meio do *link*: https://gruposdepesquisa.eerp.usp.br/sites/previnaidst/. Além disso, buscou-se adotar linguagem de fácil entendimento para a abordagem da prevenção combinada da infecção pelo HIV.

Para a apresentação da identidade visual, utilizou-se o símbolo “@” que permitiu caracterizar o aspecto informatizado, além do uso das cores azul e vermelho para representar os diferentes métodos de prevenção combinada. O *layout* de navegação foi elaborado pela equipe de pesquisadores após a definição do fluxo de acesso, dos métodos de usabilidade e dos elementos de atratividade (Figura 1). Todos os recursos utilizados visaram conduzir o usuário a uma leitura informativa, interativa, dinâmica e atualizada sobre a prevenção combinada do HIV.

**Figura 1 -:** Representação gráfica do portal PREVIN@IDST. Ribeirão Preto, SP, Brasil, 2022

O conteúdo informacional foi estruturado a partir da análise de recomendações e diretrizes clínicas, e englobou as diferentes estratégias para a prevenção combinada da infecção pelo HIV (Figura 2), que incluem as medidas voltadas para a testagem periódica, o uso consistente do preservativo, o tratamento de outras infecções, a prevenção da transmissão vertical, a imunização, a adesão à PrEP e PEP. Destaca-se a estruturação de um guia específico que considera a divulgação do tratamento do HIV com o controle de carga viral como prevenção.

O mapa, a estrutura de navegação, o fluxo de acesso e os elementos de usabilidade estão apresentados na Figura 3.

**Figura 2 -:** Infográficos e recursos de mídia adotados para a informação sobre a prevenção combinada do HIV. Ribeirão Preto, SP, Brasil, 2022

**Figura 3 -:** Mapa de navegação do portal de informação. Ribeirão Preto, SP, Brasil, 2022

Após a construção do portal, realizou-se a implementação voltada para a validação com os especialistas. Para esta etapa, participaram 24 enfermeiros com conhecimento, atuação clínica e produção científica na área de interesse e 23 especialistas em TI. Os enfermeiros eram, em sua maioria, do sexo feminino (18; 75%), com a idade média de 39,3 (DP=10,3) anos. Verificou-se a experiência dos participantes na assistência (20; 83,3%) e na docência (6; 25,1%), especialmente no ensino da Enfermagem. O tempo médio de atuação correspondeu a 8,1 anos (DP= 8,1) [0;30] e foi expressiva a frequência de participantes com produção científica na área de interesse (23; 95,8%).

Quanto aos juízes da área de TI., constatou-se a predominância do sexo masculino (13; 56,5%), com a idade média de 35,6 (DP=9,2) anos. A avaliação da formação e a atuação profissional demonstraram a experiência em diferentes áreas do *design* e do *webdesign* (9; 38,7%), por tempo médio de 8,7 anos. Ainda, destacou-se que 17 (73,9%) participantes possuíam produção científica na área.

## Indicadores de concordância e validade do PREVIN@IDST

Na avaliação de concordância pelos especialistas da saúde, para os atributos avaliados o IVC variou entre 0,90 e 1,0, e o IVC-total foi de 0,94. No atributo “avaliação da impressão geral” obteve-se IVC total de 0,96, que indicou *layout* satisfatório, estímulo à leitura, diagramação, temas, referências, fontes e cores adequadas.

Quanto aos objetivos propostos (0,90), evidenciou-se um elevado potencial para informar as pessoas que vivem com o HIV e as suas parcerias sexuais sobre a prevenção combinada. Apenas o item relacionado aos locais de busca da PrEP alcançou IVC de 0,75, levando-se em consideração a descrição geográfica dos serviços especializados em diferentes municípios do estado de São Paulo.

Na avaliação do conteúdo (0,98), todos os itens avaliados alcançaram IVC superiores a 0,90. Esses indicadores refletiram a pertinência, a utilidade e a adequação das informações ao público-alvo, além de seu uso potencial para os processos educativos em saúde e para a promoção da informação, a orientação e a prevenção combinada do HIV.

O atributo avaliado “linguagem escrita” (1,00) demonstrou clareza, objetividade, fácil assimilação e compreensão pelo público-alvo. Outros atributos avaliados foram: “atratividade” (0,91), “inovação” (0,90) e “inclusão de tópicos” (0,84). Assim, considera-se que o uso dos recursos audiovisuais, a linguagem adotada e as cores estabelecidas contribuíram para a elaboração de uma tecnologia atrativa, interessante, relevante e pertinente.

As sugestões emitidas foram acatadas e envolveram a necessidade de explorar, por meio de recursos de mídia, as estratégias de prevenção combinada. O direcionamento das medidas para os diferentes gêneros e orientações sexuais, como mulheres que fazem sexo com mulheres, foi referenciado pelos especialistas. Estão descritos os indicadores de concordância e os atributos avaliados pelos especialistas em saúde, conforme a Tabela 1.

**Tabela 1 -:** Indicadores de concordância e atributos avaliados pelos especialistas na área da saúde (n = 24). Ribeirão Preto, SP, Brasil, 2022

**Atributos**	**Concordo totalmente**	**Concordo**	**Discordo**	**Discordo totalmente**	IVC*
	**n (%)**	**n (%)**	**n (%)**	**n (%)**	
**Impressão geral**					**0,96**
Apresenta boa impressão	17(70,8)	07(29,2)	-	-	1,00
Instiga a leitura	14(58,3)	09(37,5)	-	-	1,00
Apresenta um *layout* satisfatório	12(50,0)	10(41,7)	01(4,2)	01(4,2)	0,92
Os temas abordados são adequados	18(75,0)	06(25,0)	-	-	1,00
As cores não atrapalham a leitura	15(62,5)	05(20,8)	03(12,5)	01(4,2)	0,83
A diagramação favorece o entendimento da mensagem	15(62,5)	08(33,3)	01(4,2)	-	0,96
O tamanho da letra é satisfatório para a leitura	14(58,3)	10(41,7)	-	-	1,00
As referências são pertinentes	18(75,0)	06(25,0)	-	-	1,00
**Objetivos**					**0,90**
Informa às pessoas que vivem com o HIV e suas parcerias sexuais sobre a prevenção	17(70,8)	06(25,0)	01(4,2)	-	0,96
Informa sobre a prevenção combinada do HIV	16(66,7)	08(33,3)	-	-	1,00
Representa a fonte de busca de locais para PrEP ^†^	11(45,8)	07(29,2)	05(20,8)	1(4,2)	0,75
**Conteúdo**					**0,98**
O conteúdo facilita o processo de educação em saúde na temática	20(83,3)	04(16,7)	-	-	1,00
O conteúdo permite a compreensão do tema	15(62,5)	07(29,2)	01(4,2)	-	0,96
O conteúdo obedece a uma sequência lógica	19(79,2)	05(20,8)	-	-	1,0
As orientações apresentadas foram abordadas corretamente	18(75,0)	06 (25,0)	-	-	1,0
Os termos técnicos estão adequadamente definidos	18(75,0)	04(16,7)	02(8,3)	-	0,92
As informações são satisfatórias e possuem relação com os fins desejados	18(75,0)	06(25,0)	-	-	1,0
Não existem informações desnecessárias	18(75,0)	06(25,0)	-	-	1,0
As informações são apropriadas ao público-alvo	19(79,2)	04(16,7)	01(4,2)	-	0,96
As informações são apresentadas em um contexto pertinente ao público-alvo	18(75,0)	05(20,8)	01(4,2)	-	0,96
O conteúdo é coerente com a prevenção combinada do HIV	20(83,3)	03(12,5)	01(4,2)	-	0,96
**Relevância**					**0,96**
O portal é relevante para informar a população sobre a prevenção combinada do HIV	22(91,7)	02(8,3)	**-**	**-**	1,00
As imagens e os *links* representam os aspectos importantes para o conhecimento sobre a prevenção combinada do HIV	18(75,0)	05(20,8)	01(4,2)	**-**	0,96
As imagens, os *links* e as fotografias são relevantes para o conhecimento do comportamento e das práticas de risco	16(66,7)	06(25,0)	02(8,3)	**-**	0,92
**Linguagem escrita**					**1,0**
A linguagem escrita utilizada no portal é acessível ao público-alvo	18(75,0)	6(25,0)	-	-	1,0
A linguagem verbal é de fácil assimilação	15(62,5)	9(37,5)	-	-	1,0
Os conceitos são claros e objetivos	16(66,7)	7(29,2)	-	-	1,0
**Atratividade**					**0,91**
A composição visual é atrativa e bem-organizada	12(50,0)	10(41,7)	01(4,2)	01(4,2)	0,92
São utilizados diferentes recursos audiovisuais	12(50,0)	09(37,5)	03(12,5)	-	0,88
Os diferentes recursos audiovisuais são atrativos para o portal	09(37,5)	10(41,7)	03(12,5)	01(4,2)	0,83
Possui uma linguagem atrativa	15(62,5)	09(37,5)	-	-	1,00
As imagens são interessantes	13(54,2)	09(37,5)	01(4,2)	01(4,2)	0,92
As imagens e os *links* estão integrados ao conteúdo textual	16(66,7)	07(29,2)	01(4,2)	-	0,96
As informações são pertinentes ao público-alvo	19(79,2)	04(16,7)	01(4,2)	-	0,96
O portal imprime boa impressão	19(79,2)	05(20,8)	-	-	1,00
O portal instiga a leitura	17(70,8)	06(25,0)	01(4,2)	-	0,96
As cores utilizadas não atrapalham a leitura	16(66,7)	05(20,8)	03(12,5)	-	0,88
O tamanho e o formato da letra são satisfatórios para a leitura	15(62,5)	06(25,0)	03(12,5)	-	0,88
**Inovação**					**0,90**
Possui *design* inédito	11(45,8)	10(41,7)	03(12,5)	-	0,88
Representa ideia inovadora	11(45,8)	11(45,8)	02(8,3)	-	0,92
É um meio diferente de difusão de informação	13(54,2)	10(41,7)	01(4,2)	-	0,96
É descontraído	11(45,8)	12(50,0)	01(4,2)	-	0,96
Apresenta uma linguagem que o aproxima do público	14(58,3)	10(41,7)	-	-	1,0
Aborda as informações para as diferentes orientações sexuais	16(66,7)	05(20,8)	02(8,3)	-	0,91
Aborda as informações para os diferentes gêneros	14(58,3)	05(20,8)	05(20,8)	-	0,79
Comunica com as redes sociais de uso comum	12(50,0)	09(37,5)	02(8,3)	01(4,2)	0,88
É inédito com o conteúdo sobre a prevenção combinada do HIV	11(45,8)	08(33,3)	05(20,8)	-	0,79
**IVC-Global** ^‡^					**0,94**

*IVC = Índice de Validade de Conteúdo; ^†^PrEP = Profilaxia Pré-Exposição; ^‡^IVC-Global = Índice de Validade de Conteúdo Global

Na avaliação pelos juízes da área de Informática, o portal apresentou IVC global de 0,97 (Tabela 2). A avaliação também demonstrou a qualidade de interface (0,96), a qualidade estética e audiovisual (0,99) e a facilidade de navegação (0,96). Todos os itens foram satisfatórios, conforme o referencial adotado, que indicou as evidências de validade nos índices iguais ou superiores a 0,80. Este resultado constatou que o portal apresenta os indicadores adequados de qualidade estética e audiovisual, e que o formato, a interface e o *layout* estruturados garantem facilidade e segurança para a navegação, conforme a Tabela 2.

**Tabela 2 -:** Indicadores de concordância e os atributos avaliados pelos especialistas da tecnologia da informação (n = 23). Ribeirão Preto, SP, Brasil, 2022

**Atributos**	**Concordo fortemente**	**Concordo**	**Discordo**	**Discordo fortemente**	IVC*
	**n (%)**	**n (%)**	**n (%)**	**n (%)**	
**Qualidade da interface**					**0,96**
Apresenta um *layout* satisfatório	16(69,6)	07(30,4)	-	-	1,00
O aspecto visual é bom	16(69,6)	07(30,4)	-	-	1,00
O formato das telas é satisfatório	13(56,6)	10(43,4)	-	-	1,00
Os botões de navegação são satisfatórios	15(65,2)	07(30,4)	01(4,4)	-	0,96
As cores utilizadas não atrapalham a leitura	10(43,4)	10(43,4)	02(8,8)	01(4,4)	0,87
A diagramação favorece o entendimento da mensagem	10(43,4)	12(52,2)	01(4,4)	-	0,96
A fonte escolhida é legível	12(52,1)	09(39,1)	02(8,8)	-	0,91
O tamanho da fonte escolhida é bom	08(34,8)	15(65,2)	-	-	1,00
**Qualidade estética e audiovisual**					**0,99**
A qualidade e a resolução das fotografias são satisfatórias	13(56,6)	10(43,4)	-	-	1,00
A qualidade das imagens (infográficos) é satisfatória	11(50,0)	11(50,0)	-	-	1,00
A qualidade dos *links* é satisfatória	17(73,9)	06(26,1)	-	-	1,00
A qualidade dos textos é satisfatória	13(56,6)	09(39,1)	01(4,3)	-	0,96
**Facilidade de navegação**					**0,96**
As informações são encontradas facilmente dentro do portal	12(52,2)	11(47,8)	-	-	1,00
É possível encontrar as informações sobre o portal dentro de sua própria plataforma	09(39,1)	14(60,9)	-	-	1,00
É possível encontrar as informações sobre a segurança e a privacidade dentro do portal	12(52,2)	11(47,8)	-	-	1,00
É fácil encontrar as informações sobre a segurança e a privacidade dentro do portal	07(30,5)	14(60,9)	01(4,3)	01(4,3)	0,91
É possível entrar facilmente em contato com os responsáveis pelo portal	10(43,4)	12(52,2)	01(4,4)	-	0,96
O carregamento das páginas é rápido	10(43,4)	12(52,1)	01(4,4)	-	0,96
Não há excesso de *pop-ups* (páginas flutuantes de aparecimento espontâneo) dentro do portal	14(60,9)	08(34,8)	01(4,4)	-	0,96
Não há dificuldade em acessar os *links*	13(56,6)	08(34,7)	02(8,7)	-	0,91
Há *links* diretos e funcionais para as redes sociais do portal	10(43,4)	12(52,1)	-	01(4,4)	0,96
**IVC-Global** ^†^					**0,96**

*IVC = Índice de Validade de Conteúdo; ^†^IVC-Global = Índice de Validade de Conteúdo Global

## Discussão

O portal PREVIN@IDST apresentou validade adequada entre os especialistas da área da saúde e TI. A tecnologia educacional digital pode se constituir em ferramenta contemporânea, didática, interativa e imprescindível para a informação e a popularização das estratégias para a prevenção combinada do HIV. Ainda, destaca-se o seu elevado potencial na contribuição para o alcance das metas de controles epidemiológicos propostos pelo *Joint United Nations Programme on HIV/AIDS* (UNAIDS).

A informatização da prevenção é amplamente referenciada em diferentes contextos assistenciais. Considera-se que nenhum recurso único será suficiente para controlar a epidemia e que as estratégias, quando combinadas, adaptadas ao perfil da população e incorporadas em tecnologia educacional, podem aumentar o acesso à informação. De forma igual, essa perspectiva foi apontada em estudo direcionado para o desenvolvimento de um *serious game* sobre a prática sexual segura e a contracepção para adolescentes. A proposta tecnológica, além de representar uma oportunidade para abordar o tema de maneira atraente, interativa e contextualizada à realidade do público-alvo, apresentou potencialidades para promover mudanças no comportamento e na contribuição para a informação, educação e conscientização das pessoas usuárias^(27)^.

O portal de informação configura-se como uma tecnologia educacional que está popularizada em razão de sua ampla disponibilidade, facilidade de uso e praticidade. O desenvolvimento de tecnologias em saúde para rastreio, monitoramento, prevenção, diagnóstico e tratamento das IST podem potencializar os indicadores de qualidade assistencial, o processo e o resultado clínico^(28)^. No campo de prevenção e tratamento do HIV, podem oportunizar às pessoas usuárias a possibilidade de gerar, compartilhar, interagir e receber informações, com garantia do anonimato e o sigilo de informações^(29)^.

O alto potencial para a disseminação de informações e para o alcance de populações vulnerabilizadas também constitui atributos associados à incorporação das tecnologias educacionais nas práticas de saúde. Na literatura, são evidenciadas as relações entre o nível de conhecimento com as mudanças expressivas nos comportamentos de risco, assim como com o acesso ampliado aos serviços de acolhimento e testagem, a adesão às profilaxias e às estratégias de prevenção^(28,30-31)^.

Por considerar que a eficácia das estratégias de prevenção do HIV pode variar, determinar o nível suficiente de evidência para sua inclusão na tecnologia constituiu uma etapa crítica. Nessa perspectiva, a estruturação do conteúdo foi expressa pela combinação estratégica e simultânea de intervenções biomédicas, comportamentais, estruturais, sociais e gestão de riscos que operam em múltiplos níveis.

As diferentes populações em situação de vulnerabilidade configuram-se como público-alvo das ações educativas propostas pelo PREVIN@IDST. Essa perspectiva é demonstrada pela combinação de estratégias informacionais voltadas tanto para o alcance da carga viral indetectável, quanto para a não transmissão sexual do HIV. Apesar disso, ainda existem grupos de pessoas vivendo com HIV que permanecem com risco de transmissão para suas parcerias sexuais. Assim, a diversidade populacional considerada pela tecnologia, evidencia que a prevenção do HIV será mais eficaz quando os diferentes pontos do ciclo de transmissão forem impedidos.

No mundo, as políticas públicas, as campanhas governamentais e as tecnologias para a prevenção do HIV são direcionadas fortemente para as pessoas com sorologia negativa e em situação de vulnerabilidade para a infecção. Entretanto, esse segmento compreende um grupo grande e diversificado para ser alvo de alta cobertura. Nesse sentido, projeções epidemiológicas apontam que, em 30 anos, poucos países conseguiram reverter a epidemia de HIV com base em estratégias direcionadas para pessoas soronegativas^(32)^.

Assim, a otimização da prevenção do HIV deve considerar também a alta proporção de pessoas que vivem com HIV/aids. Desse modo, os componentes potenciais da prevenção combinada do HIV além de concentrarem intervenções com eficácia comprovada ou com a indicação promissora para reduzir a suscetibilidade (o uso consistente de preservativo, a redução do risco comportamental e as profilaxias pré e pós-exposição), incorporaram nesta investigação a testagem e a adesão à TARV, o recurso terapêutico efetivo para o controle e a manutenção da carga viral em níveis indetectáveis, assim como para garantir o retardo da progressão da doença e impedir a transmissão da infecção^(8)^.

Ainda, o portal aborda a necessidade da testagem periódica para o HIV. Expandir o acesso ao conhecimento do *status* sorológico do HIV é uma prioridade global, tanto para vincular os casos positivos aos cuidados clínicos, quanto para prevenir as novas infecções. A falta de conhecimento sobre as possibilidades, a periodicidade e a indicação da testagem para o HIV, além do próprio *status* sorológico, compreendem as barreiras controle da epidemia e contribuem para a ocorrência de práticas de risco, os quais devem ser alvo de informação e conscientização popular^(32)^.

A avaliação do portal por especialistas na área em saúde contribuiu para a adequação do conteúdo, dos objetivos e da linguagem ao contexto epidemiológico, além de barreiras e desafios vivenciados no Brasil, assim como às características da população-alvo. A participação especialmente de enfermeiros nesse processo foi fundamental pelo seu protagonismo, conhecimento, competências e habilidades para a orientação, o acolhimento, a informação e a gestão de risco nas condições, nos problemas ou nas situações de saúde-doença prevalentes no cenário nacional^(21,33)^.

A validação de materiais educativos na era da informação permite a estruturação e a disponibilização do conteúdo baseado em evidências^(22)^. Ainda, promove a clareza, a objetividade e a relevância, assim como garante a atratividade ao incorporar recursos linguísticos e de mídia simples, acessíveis e de fácil assimilação. Evidências semelhantes são verificadas em outros estudos que avaliaram a linguagem de materiais educativos em saúde e que constataram que o uso de linguagem técnica compreende uma das principais barreiras no acesso à informação^(34-35)^.

O PREVIN@IDST tem potencial de inovação e informação popular. Na literatura, diferentes *websites* sobre a prevenção combinada foram desenvolvidos. Apesar disso, a maioria concentra-se na divulgação de diretrizes, protocolos e recomendações clínicas para os profissionais de saúde. A abordagem para a informação populacional ainda é limitada, incipiente e pouco explorada^(21-22)^.

O caráter inovador do portal refere-se também à qualidade da *interface*, estética e audiovisual, assim como a estruturação de uma interface para a interação e a comunicação entre os pesquisadores e as pessoas usuárias, com vistas à criação, à disponibilização e à troca de conteúdo em diferentes formatos de mídia (cartilhas, *podcast*, *e-book* e vídeos educativos).

Destaca-se que as sugestões e as críticas emitidas durante a avaliação dos especialistas contribuíram para redirecionar o recurso tecnológico aos objetivos propostos e possibilitaram minimizar vieses potenciais dos pesquisadores no processo de desenvolvimento e validação.

Como limitação deste estudo, destaca-se que os resultados desta produção demonstraram a avaliação dos atributos de validade tecnológica exclusivamente por enfermeiros. Apesar disso, destaca-se o potencial desta categoria para intervir nas condições de saúde prevalentes na população, assim como sua experiência em propor estratégias multiprofissionais para garantir a educação popular como base na promoção da saúde e prevenção de agravos. Sugerem-se estudos futuros para a mensuração dos seus efeitos no conhecimento, atitude e comportamento em saúde da população.

As implicações deste estudo para a Enfermagem e para a promoção da saúde sexual referem-se à disponibilização de uma tecnologia atrativa e gratuita, baseada em evidências de validade para a informação de diferentes populações e segmentos vulnerabilizados sobre a prevenção combinada do HIV. Ainda, destaca-se seu potencial promissor para a utilização em intervenções educacionais e de gestão de riscos, bem como para a efetivação das políticas públicas que consideram a informação e o conhecimento da doença como metas necessárias para o controle da epidemia.

## Conclusão

O PREVIN@IDST configura-se como um recurso educacional inovador e válido por reunir as evidências válidas para a informação e a orientação sobre a prevenção combinada do HIV. Os indicadores de validade destacaram a adequação do conteúdo, dos objetivos e da linguagem, assim como a boa impressão geral, facilidades de navegação, relevância, inovação, atratividade, qualidade estética e audiovisual. Ademais, destaca-se o potencial para a informação e a educação em saúde para os diferentes níveis de assistência e segmentos populacionais.

# Translation: Construcción y validación de un portal informativo sobre la prevención combinada del VIH*

**DOI:** 10.1590/1518-8345.7221.4508

**Authors:** Felipe Martins Lioi, Laelson Rochelle Milanês Sousa, Marcela Antonini, Daniel de Macedo Rocha, Henrique Ciabotti Elias, Renata Karina Reis

**Affiliations:** 1 Universidade de São Paulo, Escola de Enfermagem de Ribeirão Preto, Centro Colaborador de la OPS/OMS para el Desarrollo de la Investigación en Enfermería, Ribeirão Preto, SP, Brasil.; 2 Universidade Estadual do Maranhão, Curso de Enfermagem, Coroatá, MA, Brasil.; 3 Becaria (o) de la Coordenação de Aperfeiçoamento de Pessoal de Nível Superior (CAPES), Brasil.

**Keywords:** Estudio de Validación, VIH, Tecnologia Educacional, Prevención de Enfermedades, Acceso a la Información, Enfermería

## Abstract

construir y validar un portal de información sobre la prevención combinada de la infección por el virus de la inmunodeficiencia humana.

estudio metodológico estructurado en cuatro etapas: definición, arquitectura, diseño e implementación. El proceso de validación fue realizado por 24 enfermeros y 23 profesionales especialistas en Tecnología de la Información. Se aplicó un cuestionario tipo *Likert* para describir el nivel de concordancia entre los enfermeros/expertos y los profesionales del área de Tecnología de la Información en relación con diferentes atributos. Se consideró el Índice de Validez de Contenido con un punto de corte igual o superior a 0,80.

los índices de concordancia fueron satisfactorios, alcanzando un total global de 0,94 entre los enfermeros y de 0,96 entre los profesionales de Tecnología de la Información. Los indicadores de validez demostraron una concordancia satisfactoria en los atributos de impresión general (0,96), objetivos (0,90), adecuación del contenido (0,98) y del lenguaje (1,00), relevancia (0,96), alto potencial de atracción (0,91) e innovación (0,90). Además, la tecnología propuesta presenta recursos que garantizan facilidad de navegación (0,96), así como calidad de la interfaz (0,96), estética y elementos audiovisuales (0,99).

se concluye que el PREVI@IDST se configura como un recurso educativo innovador al reunir evidencias válidas para la información y orientación sobre la prevención combinada del VIH.

## Destacados

**(1)** El portal informativo es un recurso innovador y válido. **(2)** Reúne evidencias válidas para proporcionar información y orientación sobre la prevención del VIH. **(3)** El contenido del portal está validado. **(4)** Se trata de una nueva tecnología de educación en salud, especialmente dirigida a la Enfermería. **(5)** Puede ser utilizado en diferentes servicios de la red de atención sanitaria.

## Introducción

La infección por el Virus de la Inmunodeficiencia Humana (VIH) y el sida sigue siendo un desafío global, con impacto epidemiológico, social, cultural, económico y de salud ampliamente documentado en la literatura. Se trata de una condición crónica, emergente, progresiva y universal que, a pesar de los importantes avances, la ampliación y la efectividad de las estrategias de prevención combinada, así como la introducción y distribución gratuita de la Terapia Antirretroviral (TARV), sigue presentando notificaciones significativas tanto en países de ingresos bajos como en aquellos de ingresos medios y altos^(1-2)^.

Los datos globales estimados han demostrado la magnitud del problema. En 2023, 39,9 millones de personas vivían con VIH; se notificaron 1,3 millones de casos nuevos; 9,2 millones no recibían tratamiento y 630 mil murieron por complicaciones relacionadas con el sida^(3)^.

En Brasil, aunque se observa una tendencia decreciente en las nuevas infecciones en todas las edades y segmentos sociales, el mayor impacto epidemiológico se reporta en poblaciones clave, grupos afectados de manera desproporcionada en comparación con la población general. En esta perspectiva, se destaca la vulnerabilidad aumentada de hombres que tienen sexo con hombres, trabajadores sexuales, usuarios de drogas inyectables, personas transgénero o privadas de libertad, y que también se ve influenciada por factores socioeconómicos, prácticas de salud, y el nivel de información y conocimiento^(4-5)^.

El riesgo incrementado de infección por el VIH también se refiere a otras poblaciones como adolescentes, indígenas, mujeres embarazadas, personas negras o en situación de calle, que deben ser priorizadas debido a los contextos históricos, educativos y estructurales en los que se encuentran. Estos aspectos pueden superponer, potencializar y agravar los factores de vulnerabilidad, además de provocar cambios sustanciales en el perfil de las personas con VIH desde el inicio de la epidemia^(6)^.

Las condiciones antes mencionadas reflejan la presencia de obstáculos, barreras y desafíos asistenciales para enfrentar y controlar la epidemia de VIH/sida en Brasil. Estas limitaciones explican la alta prevalencia y la carga desproporcionada de la infección, además de restringir la percepción de vulnerabilidad, asociarse a la ocurrencia de prácticas de alto riesgo, limitar el acceso a servicios especializados de prevención y tratamiento, contribuir al diagnóstico tardío, generar un mayor impacto clínico y mantener la cascada de transmisión^(7-8)^.

En este contexto, se resalta la estrategia de prevención combinada del VIH. Esta integra múltiples estrategias de prevención para maximizar la reducción de nuevas infecciones, combinando métodos biomédicos, conductuales y estructurales basados en evidencia científica y en el respeto a los derechos humanos, adaptándose a las realidades locales. Esta estrategia ofrece diversas herramientas y opciones para que las personas elijan las que mejor se adapten a sus necesidades y preferencias, ya sea de manera individual o combinada, como el uso consistente de preservativos, pruebas diagnósticas y tratamiento de Infecciones de Transmisión Sexual (ITS), y el uso de profilaxis preexposición (PrEP) para el VIH. La prevención combinada incluye tanto la prevención primaria, dirigida a personas con serología negativa para el VIH, como las estrategias para evitar la transmisión del virus^(9-10)^.

Las estrategias biomédicas incluyen el uso de tecnologías médicas para prevenir la infección por el VIH, como PrEP, profilaxis posexposición (PEP), inmunización contra el VHB y el Virus del Papiloma Humano (VPH), tratamiento de ITS, entre otras. Por otro lado, las estrategias conductuales abarcan la realización de pruebas regulares de VIH para identificar la infección y facilitar la adhesión temprana al tratamiento, así como la participación en programas de educación en salud sexual que incrementen el conocimiento y la información sobre el virus y sus formas de prevención^(9,11)^.

Otra estrategia recomendada es la incorporación de tecnologías educativas digitales en este contexto. Sus efectos han sido explorados e indican un potencial para difundir y mejorar los mensajes de prevención, además de impactar positivamente en el nivel de conocimiento, la capacidad crítica, el proceso de toma de decisiones y la adhesión a comportamientos seguros^(11-13)^. Estas herramientas se consideran poderosas y versátiles para la creación de estrategias innovadoras que mejoren los servicios de prevención de ITS/VIH^(13-14)^.

En el ámbito de la Enfermería, las tecnologías educativas digitales en salud se configuran como métodos prometedores y atractivos que orientan las prácticas asistenciales y educativas, promoviendo una mayor adhesión al cuidado continuo, la acogida, el autocuidado, el monitoreo de condiciones de riesgo, la evaluación de resultados y la ampliación de conocimientos, actitudes y habilidades individuales y colectivas^(12,15)^. Sin embargo, aún existen lagunas en el conocimiento sobre el desarrollo de Tecnologías de la Información y la Comunicación (TIC) enfocadas en la prevención combinada del VIH, así como sobre sus efectos en el acceso al cuidado, el nivel de conocimiento, el comportamiento en salud y la información de la población.

Se sabe que una respuesta efectiva para reducir la incidencia del VIH requiere una política basada en el conocimiento, especialmente para las poblaciones más vulnerables, que tienen menor acceso a los servicios de salud^(16)^.

Ante los avances científicos y asistenciales que proponen perspectivas, iniciativas y metas para el control de la epidemia a nivel mundial, se torna fundamental la construcción y validación de tecnologías educativas en el cuidado antes de su aplicación, divulgación e incorporación en la práctica clínica, especialmente aquellas orientadas a la información sobre la prevención combinada del VIH^(10-11)^.

La desinformación y el bajo nivel de conocimiento en salud representan desafíos clave para alcanzar las metas de control de la epidemia del VIH/sida para 2030^(9)^. Por ello, promover soluciones innovadoras sobre la prevención combinada del VIH, con el potencial de llegar a las poblaciones vulnerables, constituye una necesidad urgente. En este sentido, este estudio tuvo como objetivo construir y validar un portal de información sobre la prevención combinada de la infección por VIH.

## Método

### Diseño del estudio

Se trata de un estudio metodológico estructurado en cuatro etapas de investigación: definición, arquitectura, diseño e implementación^(17-18)^. Esta investigación cumple con las recomendaciones de los *Standards for Quality Improvement Reporting Excellence* (SQUIRE 2.0), al ser una estrategia orientada a la mejora de la atención en salud.

### Lugar de recolección de datos

El estudio se llevó a cabo en una institución de educación superior ubicada en el municipio de Ribeirão Preto (SP), Brasil.

### Período

La recolección de datos se realizó entre los meses de marzo y agosto de 2022.

### Población

Participaron especialistas en salud e informática durante las etapas de elaboración y validación del portal educativo sobre la prevención combinada del VIH.

### Criterios de selección

La inclusión se basó en los criterios propuestos por Fehring^(19)^, ampliamente utilizados en estudios de validación en diversos contextos y niveles de atención en salud^(17,20-22)^. Estos criterios reflejaron la necesidad de experiencia científica, docente y/o asistencial en la prevención y enfrentamiento de la epidemia del VIH y/o en el desarrollo de tecnologías educativas. Para ello, se organizó un panel de expertos. La selección se realizó mediante consulta a la *Plataforma Lattes* del *Conselho Nacional de Desenvolvimento Científico e Tecnológico* (CNPq). Además, se consideraron investigadores registrados en la *Rede de Enfermagem Nacional em Doenças Sexualmente Transmissíveis/AIDS* (RENAIDST), procedentes de todas las regiones de Brasil.

En cuanto a los jueces del área de Tecnología de la Información (TI), participaron profesionales con experiencia en el desarrollo de tecnologías digitales, diseño y programación de *software*. El reclutamiento se llevó a cabo por correo electrónico mediante la sección de TI de la institución desarrolladora. No se aplicaron criterios específicos de exclusión, aunque se registraron pérdidas de participantes que no respondieron los cuestionarios tras tres invitaciones consecutivas o que no completaron el llenado de los instrumentos de validación.

### Participantes

La búsqueda sistematizada identificó a 36 enfermeros con potencial para inclusión. En la etapa de validación participaron 24 enfermeros con conocimiento, experiencia clínica y producción científica en el área de interés, junto con 23 especialistas en TI que respondieron el cuestionario, conformando una muestra de conveniencia. No obstante, el número de expertos incluidos cumplió con el mínimo establecido en la literatura^(23)^ y en otros estudios^(24)^.

### Variables del estudio

No hay.

### Instrumentos utilizados para la recolección de información

Se utilizó la plataforma virtual *Research Electronic Data Capture* (REDCap^®^) y se aplicó un cuestionario en línea para la recolección de datos, dividido en dos partes. La primera parte contenía variables destinadas a la caracterización sociodemográfica, laboral y educacional de los especialistas. La segunda parte consistía en preguntas que permitieron medir la concordancia en los siguientes atributos: impresión general, objetivo, contenido, relevancia, lenguaje verbal, atractivo, innovación y necesidad de incluir temas adicionales. Todos los atributos de evaluación se distribuyeron en una escala tipo *Likert* de cuatro puntos (desde “totalmente en desacuerdo” hasta “totalmente de acuerdo”).

Para los jueces del área de TI, además de la caracterización, se aplicó un cuestionario tipo *Likert* para evaluar la ergonomía e interfaz del portal educativo, así como la estructuración del diseño, diagramación, formato de pantallas, botones y recursos de navegación, calidad estética y audiovisual.

### Recolección de datos

La recolección de datos se realizó en cuatro etapas.

### Etapa 1: definición

En esta etapa se llevaron a cabo las actividades de planificación de la tecnología educativa, la definición del público objetivo, la elaboración de los recursos de navegación y la búsqueda y selección del contenido relacionado con las estrategias de prevención combinada de la infección por VIH. También se definieron los objetivos y el público objetivo, que incluyó a personas sexualmente activas, independientemente de su estado serológico para el VIH.

Durante este proceso, se realizó una búsqueda bibliográfica mediante consultas electrónicas en bases de datos, así como en manuales, guías, protocolos y recomendaciones de organismos nacionales e internacionales, con el objetivo de proporcionar un marco de referencia adecuado sobre la magnitud del problema, las estrategias de prevención y los factores asociados a una mayor vulnerabilidad de transmisión.

El contenido fue desarrollado por un grupo de enfermeros y estudiantes de posgrado con experiencia en el tema de interés y revisado por una investigadora con experiencia técnica, científica y tecnológica en el contenido. Se siguieron los principios de calidad para la elaboración de materiales educativos, los cuales consideran el uso de un lenguaje simple y accesible, así como la restricción de términos técnicos, como atributos favorables para la comprensión del contenido por parte del público objetivo^(25)^.

### Etapa 2: arquitectura

La segunda etapa se centró en la elaboración de la interfaz, los recursos visuales, la organización lógica y la estructura jerárquica del contenido. Este proceso incluyó una planificación sistematizada de la tecnología con el objetivo de definir la disposición de los elementos de consulta, el diseño estético-formal y garantizar la facilidad de comunicación e interacción con el usuario. Se buscó proponer un modelo tecnológico de fácil navegación que garantizara la accesibilidad para los usuarios^(17)^.

### Etapa 3: diseño

Se integraron los elementos de la interfaz con la composición visual para la presentación gráfica y visual del portal. Se consideró la presentación estética de la tecnología, la definición de la identidad visual, símbolos, colores, tipografía, funcionalidades, elementos de navegación, animaciones, ilustraciones, infografías, formularios, presentación textual, tablas, enlaces, sistema de etiquetas y títulos. Todas las etapas fueron desarrolladas por los investigadores y un profesional de comunicación y tecnología con amplia experiencia en la producción de materiales educativos en salud.

### Etapa 4: implementación

Se llevó a cabo la evaluación del portal de información por parte de jueces/especialistas en el área de salud y TI.

### Tratamiento y análisis de los datos

Los datos se analizaron de forma descriptiva para la caracterización de los jueces utilizando el *software Statistical Package for the Social Sciences* (SPSS), versión 25.0. El IVC (Índice de Validez de Contenido) se adoptó como parámetro de validez, considerando como satisfactorios los puntajes iguales o superiores a 0,80. El cálculo se realizó dividiendo el número de jueces que calificaron el ítem como “totalmente de acuerdo/de acuerdo” entre el número total de ítems, según lo descrito en otros estudios^(21,26)^. También se calcularon indicadores globales (IVC-global) para medir la concordancia de los jueces en relación con la representatividad de los atributos evaluados.

### Aspectos éticos

El proyecto fue aprobado por el Comité de Ética en Investigación (CEI) de la institución, proceso nº 5.068.52 el 28/10/2021. La participación fue voluntaria y estuvo condicionada a la firma del Consentimiento Informado (CI).

## Resultados

Se construyó y validó el portal de información titulado PREVIN@IDST (Figura 1). Este portal es un sitio *web* basado en Internet que aloja diversos contenidos y medios, como cartillas, videos y podcasts, y que facilita la creación de interacciones entre los investigadores y los usuarios. Además, incluye información sobre las estrategias de atención desarrolladas por los servicios de los Centros de Pruebas y Asesoramiento (CPA) y la PrEP en Brasil.

El portal tiene el potencial de alcanzar un amplio segmento de la población brasileña, dado que su acceso es gratuito mediante el enlace: https://gruposdepesquisa.eerp.usp.br/sites/previnaidst/. Asimismo, se adoptó un lenguaje de fácil comprensión para abordar la prevención combinada de la infección por VIH.

Para la identidad visual, se utilizó el símbolo “@” para representar el aspecto informatizado, junto con los colores azul y rojo para simbolizar los diferentes métodos de prevención combinada. El diseño de navegación fue desarrollado por el equipo de investigadores tras definir el flujo de acceso, los métodos de usabilidad y los elementos de atracción (Figura 1). Todos los recursos empleados se orientaron a proporcionar al usuario una experiencia de lectura informativa, interactiva, dinámica y actualizada sobre la prevención combinada del VIH.

**Figura 1 -:** Representación gráfica del portal PREVIN@IDST. Ribeirão Preto, SP, Brasil, 2022

El contenido informativo fue estructurado a partir del análisis de recomendaciones y directrices clínicas, e incluyó diversas estrategias para la prevención combinada de la infección por VIH (Figura 2). Estas estrategias abarcan medidas como la realización de pruebas periódicas, el uso consistente de preservativos, el tratamiento de otras infecciones, la prevención de la transmisión vertical, la inmunización, y la adhesión a PrEP y PEP. Se destaca la creación de una guía específica que incluye la promoción del tratamiento del VIH con el control de la carga viral como una estrategia de prevención.

El mapa, la estructura de navegación, el flujo de acceso y los elementos de usabilidad se presentan en la Figura 3.

**Figura 2 -:** Infografías y recursos multimedia adoptados para la información sobre la prevención combinada del VIH. Ribeirão Preto, SP, Brasil, 2022

**Figura 3 -:** Mapa de navegación del portal de información. Ribeirão Preto, SP, Brasil, 2022

Después de la construcción del portal, se realizó su implementación dirigida a la validación con los especialistas. En esta etapa participaron 24 enfermeros con conocimiento, experiencia clínica y producción científica en el área de interés, así como 23 especialistas en TI. La mayoría de los enfermeros eran mujeres (18; 75%), con una edad promedio de 39,3 años (DE=10,3). Se constató experiencia de los participantes en la asistencia (20; 83,3%) y en la docencia (6; 25,1%), especialmente en la enseñanza de Enfermería. El tiempo promedio de actuación fue de 8,1 años (DE=8,1) [0;30], y se observó una alta frecuencia de participantes con producción científica en el área de interés (23; 95,8%).

En cuanto a los jueces del área de TI, se observó una predominancia del sexo masculino (13; 56,5%), con una edad promedio de 35,6 años (DE=9,2). La evaluación de su formación y desempeño profesional demostró experiencia en áreas como diseño y diseño web (9; 38,7%), con un tiempo promedio de actuación de 8,7 años. Además, 17 (73,9%) de los participantes tenían producción científica en el área.

## Indicadores de concordancia y validez del PREVIN@IDST

En la evaluación de concordancia realizada por los especialistas en salud, el IVC (Índice de Validez de Contenido) para los atributos evaluados varió entre 0,90 y 1,0, con un IVC-total de 0,94.

En el atributo “evaluación de la impresión general”, el IVC total fue de 0,96, indicando un diseño visual satisfactorio, estímulo a la lectura, diagramación adecuada, temas relevantes, referencias, fuentes y colores apropiados.

Respecto a los objetivos propuestos (0,90), se evidenció un elevado potencial para informar a las personas que viven con VIH y a sus parejas sexuales sobre la prevención combinada. Sin embargo, el ítem relacionado con los lugares para buscar PrEP alcanzó un IVC de 0,75, debido a las limitaciones en la descripción geográfica de los servicios especializados en diferentes municipios del estado de São Paulo.

En la evaluación del contenido (0,98), todos los ítems alcanzaron IVC superiores a 0,90, lo que refleja la pertinencia, utilidad y adecuación de las informaciones al público objetivo, además de su potencial uso en procesos educativos en salud y para la promoción de información, orientación y prevención combinada del VIH.

El atributo “lenguaje escrito” (1,00) demostró claridad, objetividad, fácil asimilación y comprensión por parte del público objetivo. Otros atributos evaluados fueron: “atractivo” (0,91), “innovación” (0,90) e “inclusión de temas” (0,84). Se consideró que los recursos audiovisuales, el lenguaje utilizado y los colores establecidos contribuyeron a la creación de una tecnología atractiva, interesante, relevante y adecuada.

Las sugerencias emitidas fueron aceptadas e incluyeron la necesidad de explorar, mediante recursos multimedia, las estrategias de prevención combinada. Los especialistas destacaron la importancia de dirigir medidas a diferentes géneros y orientaciones sexuales, como mujeres que tienen sexo con mujeres. A continuación, se describen los indicadores de concordancia y los atributos evaluados por los especialistas en salud, según la Tabla 1.

**Tabla 1 -:** Indicadores de concordancia y atributos evaluados por los especialistas en salud (n = 24). Ribeirão Preto, SP, Brasil, 2022

**Atributos**	**Totalmente de acuerdo**	**De acuerdo**	**En Desacuerdo**	**Totalmente en desacuerdo**	IVC*
	**n (%)**	**n (%)**	**n (%)**	**n (%)**	
**Impresión general**					**0,96**
Presenta buena impresión	17(70,8)	07(29,2)	-	-	1,00
Motiva la lectura	14(58,3)	09(37,5)	-	-	1,00
Presenta un diseño satisfactorio	12(50,0)	10(41,7)	01(4,2)	01(4,2)	0,92
Los temas abordados son adecuados	18(75,0)	06(25,0)	-	-	1,00
Los colores no dificultan la lectura	15(62,5)	05(20,8)	03(12,5)	01(4,2)	0,83
La diagramación favorece la comprensión del mensaje	15(62,5)	08(33,3)	01(4,2)	-	0,96
El tamaño de la letra es satisfactorio para la lectura	14(58,3)	10(41,7)	-	-	1,00
Las referencias son pertinentes	18(75,0)	06(25,0)	-	-	1,00
**Objetivos**					**0,90**
Informa a las personas que viven con el VIH y a sus parejas sexuales sobre la prevención	17(70,8)	06(25,0)	01(4,2)	-	0,96
Informa sobre la prevención combinada del VIH	16(66,7)	08(33,3)	-	-	1,00
Representa una fuente de búsqueda de lugares para PrEP ^†^	11(45,8)	07(29,2)	05(20,8)	1(4,2)	0,75
**Contenido**					**0,98**
El contenido facilita el proceso de educación en salud sobre el tema	20(83,3)	04(16,7)	-	-	1,00
El contenido permite la comprensión del tema	15(62,5)	07(29,2)	01(4,2)	-	0,96
El contenido sigue una secuencia lógica	19(79,2)	05(20,8)	-	-	1,0
Las orientaciones presentadas han sido correctamente abordadas	18(75,0)	06 (25,0)	-	-	1,0
Los términos técnicos están adecuadamente definidos	18(75,0)	04(16,7)	02(8,3)	-	0,92
La información es satisfactoria y tiene relación con los objetivos deseados	18(75,0)	06(25,0)	-	-	1,0
No hay información innecesaria	18(75,0)	06(25,0)	-	-	1,0
La información es adecuada para el público objetivo	19(79,2)	04(16,7)	01(4,2)	-	0,96
La información se presenta en un contexto pertinente para el público objetivo	18(75,0)	05(20,8)	01(4,2)	-	0,96
El contenido es coherente con la prevención combinada del VIH	20(83,3)	03(12,5)	01(4,2)	-	0,96
**Relevancia**					**0,96**
El portal es relevante para informar a la población sobre la prevención combinada del VIH	22(91,7)	02(8,3)	**-**	**-**	1,00
Las imágenes y los enlaces representan aspectos importantes para el conocimiento sobre la prevención combinada del VIH	18(75,0)	05(20,8)	01(4,2)	**-**	0,96
Las imágenes, enlaces y fotografías son relevantes para el conocimiento de comportamientos y prácticas de riesgo	16(66,7)	06(25,0)	02(8,3)	**-**	0,92
**Lenguaje escrito**					**1,0**
El lenguaje escrito utilizado en el portal es accesible para el público objetivo	18(75,0)	6(25,0)	-	-	1,0
El lenguaje verbal es de fácil asimilación	15(62,5)	9(37,5)	-	-	1,0
Los conceptos son claros y objetivos	16(66,7)	7(29,2)	-	-	1,0
**Atracción**					**0,91**
La composición visual es atractiva y está bien organizada	12(50,0)	10(41,7)	01(4,2)	01(4,2)	0,92
Se utilizan diferentes recursos audiovisuales	12(50,0)	09(37,5)	03(12,5)	-	0,88
Los diferentes recursos audiovisuales son atractivos para el portal	09(37,5)	10(41,7)	03(12,5)	01(4,2)	0,83
Tiene un lenguaje atractivo	15(62,5)	09(37,5)	-	-	1,00
Las imágenes son interesantes	13(54,2)	09(37,5)	01(4,2)	01(4,2)	0,92
Las imágenes y los enlaces están integrados al contenido textual	16(66,7)	07(29,2)	01(4,2)	-	0,96
La información es pertinente para el público objetivo	19(79,2)	04(16,7)	01(4,2)	-	0,96
El portal causa una buena impresión	19(79,2)	05(20,8)	-	-	1,00
El portal motiva la lectura	17(70,8)	06(25,0)	01(4,2)	-	0,96
Los colores utilizados no dificultan la lectura	16(66,7)	05(20,8)	03(12,5)	-	0,88
El tamaño y el formato de la letra son satisfactorios para la lectura	15(62,5)	06(25,0)	03(12,5)	-	0,88
**Innovación**					**0,90**
Posee un diseño único	11(45,8)	10(41,7)	03(12,5)	-	0,88
Representa una idea innovadora	11(45,8)	11(45,8)	02(8,3)	-	0,92
Es un medio diferente de difusión de información	13(54,2)	10(41,7)	01(4,2)	-	0,96
Es informal y accesible	11(45,8)	12(50,0)	01(4,2)	-	0,96
Presenta un lenguaje que lo acerca al público	14(58,3)	10(41,7)	-	-	1,0
Aborda la información para diferentes orientaciones sexuales	16(66,7)	05(20,8)	02(8,3)	-	0,91
Aborda la información para diferentes géneros	14(58,3)	05(20,8)	05(20,8)	-	0,79
Se comunica con redes sociales de uso común	12(50,0)	09(37,5)	02(8,3)	01(4,2)	0,88
Es único con respecto al contenido sobre la prevención combinada del VIH	11(45,8)	08(33,3)	05(20,8)	-	0,79
**IVC-Global** ^‡^					**0,94**

*IVC = Índice de Validez de Contenido; ^†^PrEP = Profilaxis Preexposición; ^‡^IVC-Global = Índice de Validez de Contenido Global

En la evaluación realizada por los jueces del área de Informática, el portal presentó un IVC global de 0,97 (Tabla 2). La evaluación también destacó la calidad de la interfaz (0,96), la calidad estética y audiovisual (0,99) y la facilidad de navegación (0,96). Todos los ítems fueron satisfactorios, según el marco de referencia adoptado, que estableció como válidos los índices iguales o superiores a 0,80. Este resultado confirma que el portal cuenta con indicadores adecuados de calidad estética y audiovisual, y que el formato, la interfaz y el diseño estructurado garantizan la facilidad y la seguridad en la navegación, según se presenta en la Tabla 2.

**Tabla 2 -:** Indicadores de concordancia y atributos evaluados por los especialistas en tecnología de la información (n = 23). Ribeirão Preto, SP, Brasil, 2022

**Atributos**	**Totalmente de acuerdo**	**De acuerdo**	**En desacuerdo**	**Totalmente en desacuerdo**	IVC*
	**n (%)**	**n (%)**	**n (%)**	**n (%)**	
**Calidad de la interfaz**					**0,96**
Presenta un diseño satisfactorio	16(69,6)	07(30,4)	-	-	1,00
El aspecto visual es bueno	16(69,6)	07(30,4)	-	-	1,00
El formato de las pantallas es satisfactorio	13(56,6)	10(43,4)	-	-	1,00
Los botones de navegación son satisfactorios	15(65,2)	07(30,4)	01(4,4)	-	0,96
Los colores utilizados no dificultan la lectura	10(43,4)	10(43,4)	02(8,8)	01(4,4)	0,87
La diagramación favorece la comprensión del mensaje	10(43,4)	12(52,2)	01(4,4)	-	0,96
La fuente elegida es legible	12(52,1)	09(39,1)	02(8,8)	-	0,91
El tamaño de la fuente es adecuado	08(34,8)	15(65,2)	-	-	1,00
**Calidad estética y audiovisual**					**0,99**
La calidad y resolución de las fotografías son satisfactorias	13(56,6)	10(43,4)	-	-	1,00
La calidad de las imágenes (infografías) es satisfactoria	11(50,0)	11(50,0)	-	-	1,00
La calidad de los enlaces es satisfactoria	17(73,9)	06(26,1)	-	-	1,00
La calidad de los textos es satisfactoria	13(56,6)	09(39,1)	01(4,3)	-	0,96
**Facilidad de navegación**					**0,96**
La información se encuentra fácilmente dentro del portal	12(52,2)	11(47,8)	-	-	1,00
Es posible encontrar información sobre el portal dentro de su propia plataforma	09(39,1)	14(60,9)	-	-	1,00
Es posible encontrar información sobre la seguridad y la privacidad dentro del portal	12(52,2)	11(47,8)	-	-	1,00
Es fácil encontrar información sobre la seguridad y la privacidad dentro del portal	07(30,5)	14(60,9)	01(4,3)	01(4,3)	0,91
Es posible contactar fácilmente con los responsables del portal	10(43,4)	12(52,2)	01(4,4)	-	0,96
La carga de las páginas es rápida	10(43,4)	12(52,1)	01(4,4)	-	0,96
No hay un exceso de ventanas emergentes ( *pop-ups* ) dentro del portal	14(60,9)	08(34,8)	01(4,4)	-	0,96
No hay dificultad para acceder a los enlaces	13(56,6)	08(34,7)	02(8,7)	-	0,91
Hay enlaces directos y funcionales hacia las redes sociales del portal	10(43,4)	12(52,1)	-	01(4,4)	0,96
**IVC-Global** ^†^					**0,96**

*IVC = Índice de Validez de Contenido; ^†^IVC-Global = Índice de Validez de Contenido Global

## Discusión

El portal PREVIN@IDST demostró una validez adecuada entre los especialistas en salud y TI. Esta tecnología educativa digital se configura como una herramienta contemporánea, didáctica, interactiva e imprescindible para la información y la popularización de estrategias de prevención combinada del VIH. Además, destaca su alto potencial para contribuir al logro de las metas de control epidemiológico propuestas por el *Joint United Nations Programme on HIV/AIDS* (UNAIDS).

La informatización de la prevención está ampliamente referenciada en diferentes contextos asistenciales. Se considera que ningún recurso único será suficiente para controlar la epidemia y que las estrategias, cuando se combinan, se adaptan al perfil de la población e incorporan tecnologías educativas, pueden aumentar el acceso a la información. Este enfoque también se destacó en un estudio sobre el desarrollo de un *serious game* centrado en la práctica sexual segura y la anticoncepción para adolescentes. Esta propuesta tecnológica no solo representa una oportunidad para abordar el tema de manera atractiva, interactiva y contextualizada para el público objetivo, sino que también mostró potencial para promover cambios de comportamiento y contribuir a la información, educación y concienciación de los usuarios^(27)^.

El portal de información se posiciona como una tecnología educativa ampliamente popular debido a su disponibilidad, facilidad de uso y practicidad. El desarrollo de tecnologías en salud para el rastreo, monitoreo, prevención, diagnóstico y tratamiento de ITS puede mejorar los indicadores de calidad asistencial, así como los procesos y resultados clínicos^(28)^. En el ámbito de la prevención y el tratamiento del VIH, estas herramientas ofrecen a los usuarios la posibilidad de generar, compartir, interactuar y recibir información, garantizando el anonimato y la confidencialidad^(29)^.

El alto potencial para la difusión de información y el alcance a poblaciones vulnerables también son atributos asociados a la incorporación de tecnologías educativas en las prácticas de salud. En la literatura, se han evidenciado relaciones entre el nivel de conocimiento y los cambios significativos en los comportamientos de riesgo, así como un mayor acceso a servicios de acogida y pruebas, la adhesión a profilaxis y otras estrategias de prevención^(28,30-31)^.

Dado que la eficacia de las estrategias de prevención del VIH puede variar, determinar un nivel suficiente de evidencia para su inclusión en la tecnología fue una etapa crítica. Desde esta perspectiva, la estructura del contenido se diseñó mediante la combinación estratégica y simultánea de intervenciones biomédicas, conductuales, estructurales, sociales y de gestión de riesgos, operando a múltiples niveles.

Las diversas poblaciones en situación de vulnerabilidad son el público objetivo de las acciones educativas propuestas por PREVIN@IDST. Esta perspectiva se refleja en la combinación de estrategias informativas destinadas tanto al logro de una carga viral indetectable como a la prevención de la transmisión sexual del VIH. Sin embargo, aún existen grupos de personas que viven con VIH que permanecen en riesgo de transmisión hacia sus parejas sexuales. Por lo tanto, la diversidad poblacional considerada en esta tecnología demuestra que la prevención del VIH será más eficaz cuando se bloqueen los diferentes puntos del ciclo de transmisión.

A nivel mundial, las políticas públicas, las campañas gubernamentales y las tecnologías de prevención del VIH se dirigen principalmente a personas con serología negativa y en situación de vulnerabilidad ante la infección. Sin embargo, este grupo es amplio y diverso, lo que dificulta una cobertura alta y efectiva. Proyecciones epidemiológicas indican que, en 30 años, pocos países han logrado revertir la epidemia de VIH basándose únicamente en estrategias dirigidas a personas seronegativas^(32)^.

Por ello, la optimización de la prevención del VIH debe incluir también a la alta proporción de personas que viven con VIH/sida. Los componentes potenciales de la prevención combinada del VIH no solo incluyen intervenciones con eficacia comprobada, como el uso consistente del preservativo, la reducción del riesgo conductual y las profilaxis pre y posexposición, sino que también incorporan pruebas diagnósticas y la adhesión a la TARV. Este recurso terapéutico es efectivo para controlar y mantener la carga viral en niveles indetectables, retrasar la progresión de la enfermedad e impedir la transmisión de la infección^(8)^.

El portal también aborda la necesidad de realizar pruebas periódicas para el VIH. Expandir el acceso al conocimiento del estado serológico del VIH es una prioridad global, tanto para vincular los casos positivos a los cuidados clínicos como para prevenir nuevas infecciones. La falta de conocimiento sobre las posibilidades, la periodicidad y las indicaciones de las pruebas de VIH, además del desconocimiento del propio estado serológico, constituyen barreras para controlar la epidemia y contribuyen a la práctica de comportamientos de riesgo, los cuales deben ser objetivos de información y concienciación pública^(32)^.

La evaluación del portal por especialistas en el área de la salud contribuyó a la adecuación del contenido, los objetivos y el lenguaje al contexto epidemiológico, así como a las barreras y desafíos presentes en Brasil, y a las características de la población objetivo. La participación de enfermeros en este proceso fue fundamental debido a su protagonismo, conocimiento, competencias y habilidades para la orientación, acogida, información y gestión de riesgos en condiciones, problemas o situaciones de salud-enfermedad prevalentes en el escenario nacional^(21,33)^.

La validación de materiales educativos en la era de la información permite estructurar y disponibilizar contenidos basados en evidencias^(22)^. Además, promueve la claridad, la objetividad y la relevancia, al mismo tiempo que garantiza la atracción mediante la incorporación de recursos lingüísticos y multimedia simples, accesibles y de fácil comprensión. Evidencias similares se han verificado en otros estudios que evaluaron el lenguaje de materiales educativos en salud, concluyendo que el uso de lenguaje técnico constituye una de las principales barreras para el acceso a la información^(34-35)^.

El PREVIN@IDST tiene potencial innovador y de popularización de la información. En la literatura, diversos sitios *web* sobre prevención combinada han sido desarrollados. Sin embargo, la mayoría se enfoca en la divulgación de directrices, protocolos y recomendaciones clínicas dirigidas a profesionales de la salud. La información orientada a la población general sigue siendo limitada, incipiente y poco explorada^(21-22)^.

El carácter innovador del portal también radica en la calidad de su interfaz, estética y recursos audiovisuales, así como en la estructuración de una plataforma interactiva para la comunicación entre investigadores y usuarios. Esto permite la creación, disponibilidad e intercambio de contenido en diversos formatos multimedia, como cartillas, podcasts, libros electrónicos y videos educativos.

Cabe destacar que las sugerencias y críticas emitidas durante la evaluación por los especialistas contribuyeron a redirigir el recurso tecnológico hacia los objetivos propuestos, minimizando posibles sesgos de los investigadores en el proceso de desarrollo y validación.

Como limitación de este estudio, se señala que los resultados reflejan la evaluación de los atributos de validez tecnológica exclusivamente por parte de enfermeros. No obstante, se resalta el potencial de esta categoría profesional para intervenir en las condiciones de salud prevalentes en la población, así como su experiencia en proponer estrategias multiprofesionales para garantizar la educación popular como base para la promoción de la salud y la prevención de enfermedades. Se sugieren futuros estudios para medir sus efectos en el conocimiento, actitudes y comportamientos de salud en la población.

Las implicaciones de este estudio para la enfermería y la promoción de la salud sexual incluyen la disponibilización de una tecnología atractiva y gratuita, basada en evidencias de validez, destinada a informar a diferentes poblaciones y segmentos vulnerables sobre la prevención combinada del VIH. Además, se destaca su potencial prometedor para su uso en intervenciones educativas y de gestión de riesgos, así como para la implementación de políticas públicas que consideren la información y el conocimiento de la enfermedad como metas necesarias para el control de la epidemia.

## Conclusión

El PREVIN@IDST se configura como un recurso educativo innovador y válido al reunir evidencias sólidas para la información y orientación sobre la prevención combinada del VIH. Los indicadores de validez destacaron la adecuación del contenido, los objetivos y el lenguaje, así como una buena impresión general, facilidad de navegación, relevancia, innovación, atractivo, calidad estética y audiovisual. Además, se resalta su potencial para la información y educación en salud en los diferentes niveles de atención y segmentos poblacionales.
